# The global burden of disease attributable to preterm birth and low birth weight in 204 countries and territories from 1990 to 2019: An analysis of the Global Burden of Disease Study

**DOI:** 10.7189/jogh.14.04109

**Published:** 2024-07-12

**Authors:** Taixiang Liu, Yanping Xu, Yanfeng Gong, Jinxin Zheng, Zheng Chen

**Affiliations:** 1 Department of Neonatal Intensive Care Unit, The Children’s Hospital, Zhejiang University School of Medicine, Hangzhou, China; 2 National Clinical Research Centre for Child Health, Hangzhou, China; 3 Fudan University School of Public Health, Shanghai, China; 4 School of Global Health, Chinese Centre for Tropical Diseases Research, Shanghai Jiao Tong University School of Medicine, Shanghai, China; 5 One Health Centre, Shanghai Jiao Tong University/The University of Edinburgh, Shanghai, China

## Abstract

**Background:**

Preterm birth and low birth weight (PBLBW), recognised globally as primary contributors to infant mortality in children under five, have not been sufficiently investigated in terms of their worldwide impact. In this study we aimed to thoroughly evaluate the contemporary trends in disease burden attributable to PBLBW.

**Methods:**

We analysed data from 204 countries and territories between 1990–2019, as sourced from the 2019 Global Burden of Disease Study. We analysed the global incidence of mortality and disability-adjusted life years (DALYs) associated with PBLBW, stratified by age, gender, year, and geographic location, alongside the socio-demographic index (SDI). We calculated the annual percentage changes to evaluate the dynamic trends over time. We employed a generalised linear model and scrutinised the relationship between the SDI and the disease burden attributed to PBLBW.

**Results:**

In 2019, the global age-standardised rate of deaths and DALYs related to PBLBW showed significant declines. Over the period 1990–2019, both death and DALY rates displayed substantial downward trends, with similar change trends observed for both females and males. Age-specific ratios revealed a decrease in PBLBW-related deaths and DALYs with increasing age, primarily during the neonatal stages (zero to 27 days). The leading three causes of PBLBW-related DALYs in 2019 were neonatal disorders, lower respiratory infections, and sudden infant death syndrome. Furthermore, the association between SDI and PBLBW-related DALYs indicated that the age-standardised DALY rates in 204 countries and territories worldwide were negatively correlated with SDI in 2019. From 1990 to 2019, the age-standardised DALY rates decreased linearly in most regions, except sub-Saharan Africa.

**Conclusions:**

The persistent global burden of disease associated with PBLBW is particularly pronounced in neonates aged less than 28 days and in regions with low SDI. In this study, we highlighted the critical need for tailored interventions aimed at mitigating the detrimental effects of PBLBW to attain specific sustainable development goals, particularly those centred on enhancing child survival and overall well-being.

Preterm birth and low birth weight (PBLBW) persist as critical public health concerns worldwide, significantly contributing to the global disease burden [1]. Preterm birth (PTB) is characterised by the delivery of an infant before reaching 37 weeks of gestation. Low birth weight (LBW) is defined as a birth weight of less than 2500 g, irrespective of the duration of gestation [2,3]. Both conditions are intricately linked with elevated rates of morbidity and mortality in neonates and present substantial long-term health challenges. This association underscores the need for continued research and intervention strategies to effectively address these prevalent health issues.

Recent data indicate that PTB impacts over 15 million infants annually, representing approximately 11% of all live births globally [1]. Notably, the prevalence of PTB exhibits significant geographical variation, with disproportionately higher rates in low- and middle-income countries [4]. LBW affects an estimated 20 million newborns annually, intensifying the global health burden [2]. The implications of PBLBW extend far beyond the neonatal phase. Individuals born preterm or with LBW are at an increased risk for a spectrum of health issues spanning their lifetime [5,6]. These complications include, but are not limited to, respiratory distress syndrome, necrotic enterocolitis, cognitive impairments, developmental delays, sensory deficits such as impaired vision and hearing, and heightened susceptibility to chronic conditions like cardiovascular diseases and chronic kidney disease [7–13].

The epidemiological understanding of diseases associated with PBLBW is fundamental for informed health care planning, effective resource allocation, and the development of precise interventions. Quantifying the burden of diseases attributable to PBLBW is critical, enabling policymakers and health care practitioners to formulate strategies to alleviate its impact on individuals, families, and communities. Despite the significance of this issue, there is a notable scarcity of comprehensive, population-based studies that assess the global burden of PBLBW. In response to this gap, we aimed to analyse global mortality and disability-adjusted life years (DALYs) associated with PBLBW, drawing upon data from the 2019 Global Burden of Disease (GBD) study. This research provides essential insights and data for shaping public health interventions geared towards diminishing the prevalence and impact of diseases related to PBLBW.

## METHODS

### Data sources

The data used in this study were obtained from the GBD 2019 database [14]. The GBD project is an international research initiative that spans multiple countries and regions. It utilises a standardised methodology to estimate and analyse the burden of 369 diseases or injuries and 87 risk factors in 204 countries and territories worldwide since 1990, categorised by age, year, and gender [15–17]. GBD collaborators collected relevant data from field surveys, population censuses, life statistics, and other health-related data sources. Details about the data, methods, and statistical modelling used in GBD 2019 and the comparative risk assessment specifically for PBLBW have been described elsewhere [4]. Based on the sociodemographic index (SDI), 204 countries or regions were divided into five regions, and they were further categorised into 21 GBD regions according to epidemiological characteristics and geographical proximity [15–17]. The GBD database is maintained by the Institute for Health Metrics and Evaluation (IHME) and is publicly accessible. This study complies with the Guidelines for Accurate and Transparent Health Estimates Reporting (GATHER) statement [18].

### Definitions

GBD 2019 utilised the Bayesian regression tool DisMod-MR, version 2.1 (Institute for Health Metrics and Evaluation, Washington D.C., USA) to analyse, model, and estimate deaths, death rates, DALYs, and DALY rates as indicators for quantifying the burden attributed to PBLBW, which is defined as newborns with a gestational age of less than 37 weeks and a birth weight of less than 2500 g. DALY represents the total years of healthy life lost due to disease, consisting of years lived with disability and years of life lost due to premature death [19]. Age-standardised rates (ASR) were calculated using the average age distribution of the world population for the period 2010–35 as the standard. All rates were reported per 100 000 population, resulting in standardised death rates and standardised DALY rates [20]. Additionally, the study employed the population-attributable fraction approach, comparing the exposure levels of the target population to the theoretical minimum risk exposure level while assuming that the exposure levels of other risk factors remain constant [19]. This allowed estimation of the extent of harm caused by risk factor exposures in the population affected by PBLBW.

Age groups ranged from zero to six days, seven to 27 days, 28–364 days, one to four years, five to nine years, 10–19 years, and 20+ years, totalling seven age groups. SDI is a composite index reflecting the status of social demographic development at the country/region level. It is derived from data such as the total fertility rate among females <25 years, the average education level of females aged ≥15 years, and per capita income. SDI ranges from zero to one, where zero indicates the theoretical minimum level of development associated with health outcomes in the region, and one represents the theoretical maximum level of development [21]. GBD 2019 classified countries/regions into five development quintiles based on their SDI values – low, low-middle, middle, high-middle, and high [21].

### Statistical analysis

In this study, we calculated the burden of disease associated with PBLBW, encompassing death counts, DALYs, and their ASR. These were accompanied by a percent change analysis, all presented with 95% uncertainty intervals (UIs). For ASR, we employed global age structure standardisation, a crucial approach for fair comparisons across diverse populations or timeframes. During the modelling process, we meticulously addressed parameter uncertainty by randomly sampling 1000 times from specific age, gender, location, and year distributions and propagating this uncertainty throughout the analysis [22]. Additionally, 95% UIs for final estimates were calculated using 1000 random samples, determining the bounds by the 25th and 975th percentiles. This approach ensured a robust estimation process that accounts for uncertainty [22]. Moreover, we quantified the attributable fraction of age-standardised DALYs to PBLBW using a population-attributable fraction. This metric indicates the potential reduction in DALYs under an ideal exposure scenario. For these estimates, we utilised the GBD 2019 comparative risk assessment methodology.

In addition, to quantify the evolving trends, we computed the estimated annual percentage change for age-standardised deaths and DALYs specifically linked to PBLBW. Estimated annual percentage change, a widely adopted metric, provides a comprehensive overview of changes in ASR across a designated timeframe. We employed a regression analysis, fitting a line to the natural logarithm (ln) of these rates, expressed as:

y = α + βx + ε

where y is the ln (ASR), α is the constant term, β determines the negative or positive trends of the selected ASR, x is the calendar year, and ε is the error term. Subsequently, estimated annual percentage change was derived using the formula 100 × (e^β^ – 1), and its associated 95% confidence interval (CI) was determined to accurately reflect the temporal pattern in ASR [4].

We employed the generalised linear model to analyse the relationship between DALYs and death rates in PBLBW, along with various sociodemographic factors. The specifics are outlined as follows: the ln of DALYs or death rates was regressed against a set of covariates, including gross domestic product (GDP), current health expenditure as a percentage of GDP, the proportion of the population aged ≥60 years, the urban population percentage, and population density [23]. The selection of the most appropriate model was based on the Akaike information criterion, ensuring a robust and accurate fit [23]. Finally, our analysis explored the correlation between the SDI and the PBLBW-related disease burden, disaggregated by location and year.

## RESULTS

### The burden caused by PBLBW globally

From 1990 to 2019, there was a notable decline in deaths and DALYs attributable to PBLBW for both females and males globally (Table S1 in the **Online Supplementary Document**). The number of global PBLBW-related deaths decreased from 3.2 million (95% UI = 3.0, 3.4) to 1.8 million deaths (95% UI = 1.5, 2.1), and DALYs decreased from 286.5 million (95% UI = 266.6, 307.1) to 170.1 million (95% UI = 145.9, 201.3) in 2019. Specifically, the number of global PBLBW-related deaths in males fell from 1.8 million (95% UI = 1.7, 1.9) to 1.0 million (95% UI = 0.9, 1.2), and DALYs decreased from 162.0 million (95% UI = 149.9, 174.6) to 96.6 million (95% UI = 82.1, 114.6). For females, PBLBW-related global deaths dropped from 1.4 million (95% UI = 1.3, 1.5) to 0.8 million (95% UI = 0.7, 0.9), while DALYs reduced from 124.4 million (95% UI = 115.4, 134.2) to 73.5 million (95% UI = 63.6, 85.8). Throughout the period 1990–2019, the ASR of PBLBW-related deaths and DALYs decreased by 42.2% (–42.8% for females and –41.9% for males) and 40.5% (–40.8% for females and –40.2% for males) (**Table 1**).

**Table 1:** Age-standardised deaths and DALYs attributable to PBLBW in 2019 and percentage change from 1990 to 2019 by sex, global, and SDI regions

Region and sex	Deaths						DALYs					
	1990 ASR, n (95% UI)*	2019 ASR, n (95% UI)*	**Percentage change in ASR (1990–2019), % (95% CI)**	**1990 ASR PAF, n (95% UI)**	**2019 ASR PAF, n (95% UI)**	**Percentage change in ASR PAF (1990–2019), % (95% CI)**	1990 ASR, n (95% UI)*	2019 ASR, n (95% UI)*	**Percentage change in ASR (1990–2019), % (95% CI)**	**1990 ASR PAF, n (95% UI)**	**2019 ASR PAF, n (95% UI)**	**Percentage change in ASR PAF (1990–2019), % (95% CI)**
Global												
*Both*	48.3 (44.9, 51.8)	27.9 (23.7, 33.1)	–42.2 (50.9, –31.6)	4.3 (4.1, 4.6)	3.8 (3.3, 4.3)	–12.6 (–24.1, 0.8)	4376.5 (4073.2, 4688.6)	2604.4 (2232.2, 3085.7)	–40.5 (–49.0, –29.9)	8.7 (8.1, 9.4)	7.9 (6.9, 9.0)	–9.4 (–19.5, 2.2)
*Female*	43.2 (40.1, 46.6)	24.7 (21.2, 29.1)	–42.8 (–51.0, –32.8)	4.5 (4.2, 4.8)	4.0 (3.5, 4.6)	–11.0 (–23.0, 1.9)	3930.8 (3647.4, 4239.7)	2325.7 (2008.5, 2712.1)	–40.8 (–48.9, –30.9)	8.5 (7.7, 9.3)	7.7 (6.6, 8.8)	–9.3 (–19.2, 2.1)
*Male*	53.0 (49.0, 57.2)	30.8 (25.9, 37.0)	–41.9 (–51.1, –30.1)	4.1 (3.8, 4.4)	3.5 (3.0, 4.1)	–13.2 (–25.6, 1.7)	4793.7 (4432.6, 5163.4)	2864.4 (2435.1, 3403.9)	–40.2 (–49.5, –28.7)	8.9 (8.2, 9.5)	8.0 (6.9, 9.2)	–9.3 (–20.2, 3.0)
High SDI												
*Both*	8 (7.5, 8.5)	3.7 (3.3, 4.1)	–54.1 (–59.3, –48.4)	1.2 (1.1, 1.2)	0.8 (0.7, 0.9)	–30.6 (–38.0, –22.8)	826.7 (779.9, 879.4)	451.6 (408.6, 497.9)	–45.4 (–50.5, –40.3)	3.0 (2.7, 3.4)	2.1 (1.8, 2.5)	–30.0 (–36.3, –23.6)
*Female*	7.0 (6.6, 7.5)	3.3 (3.0, 3.7)	–52.8 (–58.2, –47.1)	1.3 (1.2, 1.4)	0.9 (0.8, 1.0)	–29.5 (–37.2, –21.7)	743.3 (695.3, 796.2)	421.7 (380.3, 464.2)	–43.3 (–48.8, –38.3)	3.1 (2.7, 3.6)	2.1 (1.8, 2.5)	–31.3 (–37.6, –25.3)
*Male*	8.9 (8.4, 9.5)	4.0 (3.5, 4.5)	–55.1 (–61.4, –48.9)	1.0 (1.0, 1.1)	0.7 (0.6, 0.8)	–29.3 (–38.6, –20.0)	905.1 (849.4, 966.4)	479.7 (427.0, 534.7)	–47.0 (–52.6, –41.1)	2.9 (2.6, 3.2)	2.1 (1.8, 2.4)	–27.8 (–35.1, –20.0)
High-middle SDI												
*Both*	22.8 (20.9, 24.9)	7.1 (6.1, 8.3)	–68.8 (–74.1, –62.6)	2.4 (2.2, 2.6)	1.2 (1.0, 1.3)	–50.7 (–58.8, –41.8)	2118.8 (1941.8, 2297.9)	748.9 (654.7, 864.1)	–64.7 (–70.0, –58.8)	5.8 (5.2, 6.4)	3.1 (2.7, 3.7)	–45.9 (–53.4, –37.5)
*Female*	20.0 (18.3, 21.7)	6.5 (5.6, 7.5)	–67.5 (–72.7, –61.6)	2.6 (2.3, 2.8)	1.4 (1.2, 1.6)	–47.0 (–55.5, –36.9)	1870.7 (1720.6, 2026.3)	694.3 (611.9, 793.5)	–62.9 (–68.0, –56.9)	5.9 (5.2, 6.7)	3.3 (2.8, 3.9)	–44.0 (–51.5, –35.7)
*Male*	25.5 (23.3, 28.0)	7.7 (6.5, 9.1)	–69.7 (–75.3, –63.4)	2.1 (1.9, 2.3)	1.0 (0.8, 1.2)	–52.9 (–61.3, –43.5)	2347.7 (2146.4, 2570.1)	798.4 (692.6, 928.7)	–66.0 (–71.4, –59.8)	5.5 (5.0, 6.1)	2.9 (2.5, 3.4)	–47.0 (–55.3, –38.2)
Middle SDI												
*Both*	35.7 (33.2, 38.3)	15.4 (13.0, 18.2)	–57.0 (–63.8, –48.7)	3.2 (3.0, 3.5)	2.1 (1.8, 2.5)	–35.3 (–45.0, –23.2)	3252.1 (3028.5, 3482.2)	1488 (1272.5, 1744.6)	–54.2 (–61.0, –46.0)	7.3 (6.7, 7.9)	5.2 (4.4, 6.0)	–28.8 (–38.1, –17.5)
*Female*	31.4 (29.2, 33.7)	13.7 (11.7, 16.2)	–56.4 (–62.9, –48.0)	3.2 (3.0, 3.5)	2.3 (1.9, 2.6)	–29.7 (–40.2, –15.8)	2873.1 (2674.5, 3076.1)	1342.2 (1161.2, 1564.1)	–53.3 (–59.9, –44.9)	6.9 (6.3, 7.6)	5.2 (4.4, 6.0)	–25.2 (–34.8, –13.4)
*Male*	39.7 (36.6, 42.9)	16.9 (14.1, 20.2)	–57.5 (–64.6, –48.9)	3.2 (2.9, 3.5)	1.9 (1.6, 2.3)	–39.8 (–49.8, –27.9)	3604.1 (3320.3, 3895.7)	1621.8 (1378.3, 1917.0)	–55.0 (–62.2, –46.2)	7.5 (6.9, 8.2)	5.1 (4.3, 6.0)	–32.0 (–41.4, –20.4)
Low-middle SDI												
*Both*	73.2 (67.9, 78.9)	38.3 (32.5, 44.9)	–47.7 (–56.0, –38.1)	5.0 (4.7, 5.3)	4.0 (3.5, 4.6)	–18.9 (–30.5, –6.4)	6597.3 (61154, 7103.9)	3551.5 (3040.3, 4157.8)	–46.2 (–54.5, –36.6)	10.2 (9.5, 10.9)	9.0 (7.8, 10.2)	–11.3 (–22.0, 0.3)
*Female*	66.9 (60.8, 73.3)	35.0 (30.0, 40.8)	–47.7 (–56.0, –37.9)	5.0 (4.5, 5.4)	4.2 (3.6, 4.7)	–15.9 (–28.7, –2.1)	6034.6 (5493.7, 6600.7)	3258.1 (2813.4, 3775.2)	–46.0 (–54.4, –36.2)	9.6 (8.7, 10.5)	8.8 (7.6, 10.0)	–8.7 (–20.2, 4.2)
*Male*	79.1 (72.8, 85.8)	41.4 (34.7, 49.1)	–47.8 (–56.9, –37.5)	5.0 (4.6, 5.4)	3.9 (3.3, 4.5)	–22.0 (–34.9, –8.7)	7125.5 (6563.1, 7714.3)	3825.2 (3229.1, 4518.6)	–46.3 (–55.4, -36.2)	10.6 (9.9, 11.5)	9.2 (7.8, 10.5)	–13.9 (–25.7, –1.3)
Low SDI												
*Both*	73.9 (67.8, 80.3)	45.4 (37.4, 55.3)	–38.7 (–48.7, –25.3)	4.1 (3.8, 4.4)	4.0 (3.4, 4.6)	–3.2 (–17.2, 11.9)	6630.2 (6079.4, 7199.4)	4141.7 (3429.3, 5019.0)	–37.5 (–47.5, –24.4)	7.9 (7.3, 8.5)	8.4 (7.4, 9.5)	6.4 (–5.7, 19.6)
*Female*	65.2 (59.7, 71.0)	38.9 (32.5, 46.5)	–40.4 (–49.4, –28.2)	3.9 (3.7, 4.2)	3.8 (3.2, 4.3)	–5.0 (–18.2, 8.9)	5852.0 (5355.1, 6364.2)	3561.8 (2997.9, 4245.9)	–39.1 (–48.2, –27.1)	7.3 (6.7, 7.9)	7.6 (6.7, 8.6)	4.9 (–7.0, 17.3)
*Male*	82.3 (75.0, 90.0)	51.5 (41.9, 63.6)	–37.3 (–48.0, –23.2)	4.2 (3.9, 4.5)	4.1 (3.5, 4.8)	–2.5 (–18.1, 13.9)	7372.0 (6727.6, 8059.6)	4694.0 (3842.3, 5758.3)	–36.3 (–46.9, –22.3)	8.4 (7.8, 9.1)	9.0 (7.9, 10.3)	7.1 (–6.1, 21.3)

ASR – age-standardised rates, DALY – disability-adjusted life year, PAF – population-attributable fraction, PBLBW – preterm birth and low birth weight, SDI – socio-demographic index

*Per 100 000 population.

**Figure 1:**
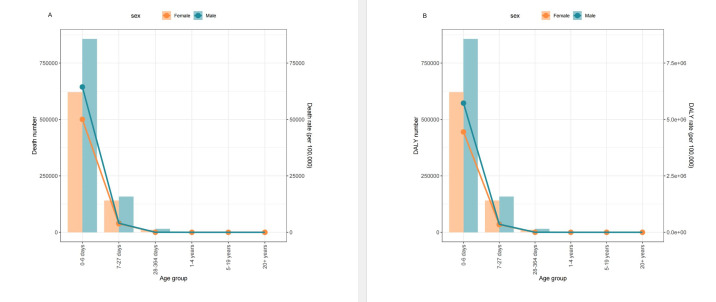
Age-specific numbers and rates of deaths and DALYs attributable to preterm birth and low birth weight by sex in 2019. **Panel A.** Deaths. **Panel B.** DALYs. DALY – disability-adjusted life year.

The age-specific ratios for PBLBW-related deaths and DALYs showed a decrease with increasing age, occurring primarily during the neonatal stages, with a similar pattern observed for both females and males (**Figure 1**). Moreover, the highest numbers of deaths and DALYs, as well as deaths and DALY rates associated with PBLBW, were found in the age group zero to six days. For the age group under 365 days, the number of PBLBW-related deaths and DALYs, deaths and DALY rates were higher for males than for females of the same age group. Beyond 365 days, the numbers related to PBLBW between males and females were roughly equivalent.

In 2019, across the 21 GBD regions, western sub-Saharan Africa experienced the highest ASR for deaths related to PBLBW, reaching 50.6 deaths per 100 000 population (95% UI = 42.0, 61.4). South Asia had the highest ASR for DALYs associated with PBLBW, standing at 7388.4 DALYs per 100 000 population (95% UI = 6764.9, 8071.0). In the high-income Asia Pacific region, the occurrence rates for PBLBW-related deaths and DALYs were the lowest. There were 1.3 deaths per 100 000 population (95% UI = 1.1, 1.4), and 468.5 DALYs per 100 000 population (95% UI = 423.3, 530.5). In the period 1990–2019, the greatest percentage decrease in PBLBW-related deaths and DALY rates occurred in East Asia. For deaths, the percentage decrease was 77.8% (95% UI = –81.1, –72.7), and for DALYs 74.0% (95% UI = −77.8, −69.3) (Table S2 in the **Online Supplementary Document**).

**Figure 2:**
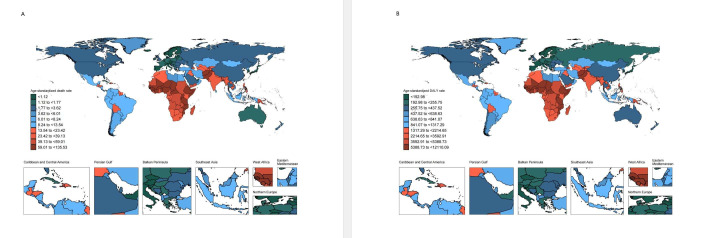
Age-standardised death and DALY rates attributable to preterm birth and low birth weight in 2019. **Panel A.** Deaths. **Panel B.** DALYs. DALY – disability-adjusted life year.

In 2019, Pakistan recorded the highest ASR of deaths and DALYs attributed to PBLBW. Specifically, the ASR for deaths stood at 69.4 per 100 000 population (95% UI = 56.6, 83.8), while the ASR for DALYs was 6301.6 per 100 000 population (95% UI = 5168.6, 7591.5). In contrast, Japan had the lowest rates, with an ASR of 0.9 deaths per 100 000 population (95% UI = 0.7, 1.0) and 156.5 DALYs per 100 000 population (95% UI = 134.4, 180.3) (**Figure 2**, Table S3 in the **Online Supplementary Document**). In the period 1990–2019, the Cook Islands experienced the most significant decline in both the death rate and DALYs related to PBLBW, with a decrease of 93.0% in the death rate (95% UI = –95.6, –90.0) and 84.4% in DALYs (95% UI = –88.2, –80.1). Conversely, among all countries and regions, Guam witnessed the highest increase (30.7%) in PBLBW-related deaths (95% UI = –4.1, 73.6) and 28.2% DALY rates (95% UI = –2.3, 64.6).

### Impact of PBLBW on specific diseases

In 2019, neonatal disorders were the predominant cause of DALYs associated with PBLBW, resulting in 2324.3 DALYs per 100 000 population (95% UI = 1990.7, 2734.1) (Table S4 in the **Online Supplementary Document**). Neonatal disorders accounted for 82.2% of the total age-standardised DALYs related to PBLBW (95% UI = 80.6, 83.7). Following neonatal disorders, the next leading causes were lower respiratory infections (LRIs) (15.5%), sudden infant death syndrome (SIDS) (7.6%), and meningitis (6.4%). A similar pattern was also noted in mortality rates, where neonatal disorders represented 85.1% of all age-standardised deaths linked to PBLBW.

**Figure 3:**
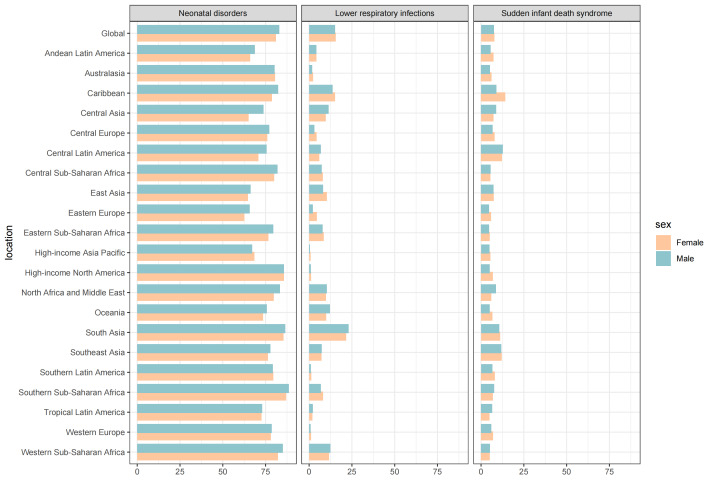
Fraction of neonatal disorders, lower respiratory infections and sudden infant death syndrome age-standardised death DALYs attributable to PBLBW by region for females and males in 2019. The three leading GBD-level three causes of PBLBW-related DALYs are shown. DALY – disability-adjusted life year, PBLBW – preterm birth and low birth weight, GBD – Global Burden of Disease Study.

There were significant disparities in the proportions of PBLBW-related age-standardised DALYs that could be attributed to specific regions (**Figure 3**). Across the 21 GBD regions in 2019, southern sub-Saharan Africa recorded the highest attributable proportions of age-standardised DALYs due to PBLBW for neonatal disorders in males. Eastern Europe had the lowest attributable proportions for neonatal disorders in females. In the Caribbean, females had the highest attributable proportions for level three GBD causes like diarrheal diseases, meningitis, and SIDS. In high-income Asia Pacific, males had the lowest attributable proportions for level three GBD causes such as LRIs and otitis media (Table S5 in the **Online Supplementary Document**).

### Relationship between SDI and PBLBW on disease burden

In 2019, among countries with the lowest SDI, the ASR of deaths related to PBLBW was 45.4 per 100 000 individuals (95% UI = 37.4, 55.3). The corresponding ASR of DALYs attributed to PBLBW was 4141.7 per 100 000 individuals (95% UI = 3429.3, 5019.0). In contrast, countries in the highest SDI quintile exhibited significantly lower ASRs for PBLBW-related deaths and DALYs, with 3.7 deaths per 100 000 individuals (95% UI = 3.3, 4.1) and 451.6 DALYs per 100 000 individuals (95% UI = 408.6, 497.9), respectively. From 1990 to 2019, the highest percentage changes in age-standardised population-attributable fraction of deaths and DALYs attributable to PBLBW were observed in the high-middle SDI quintile (**Table 1**).

**Figure 4:**
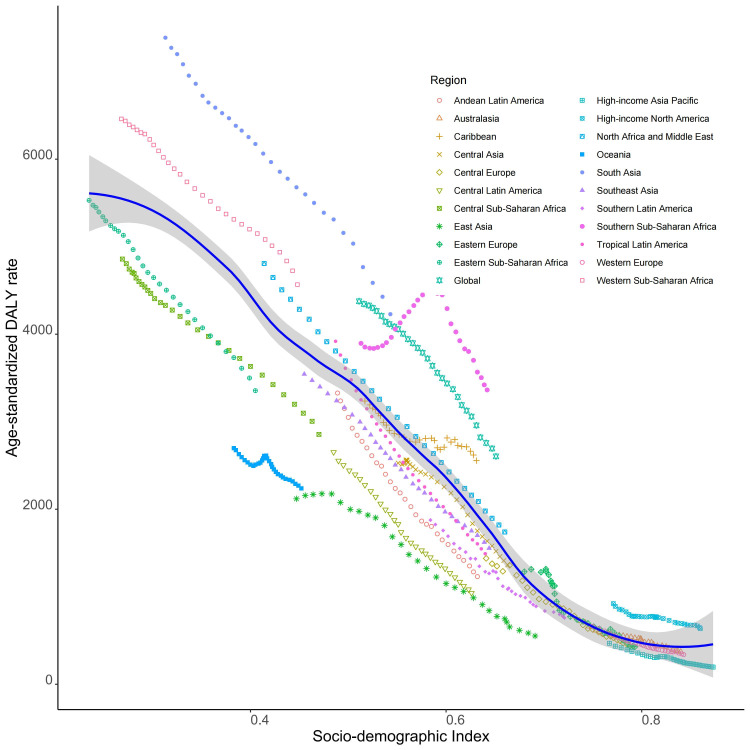
Age-standardised DALY rates attributable to preterm birth and low birth weight across 21 GBD regions by socio-demographic index (1990–2019). For each region, points from left to right depict estimates from each year from 1990 to 2019. DALY – disability-adjusted life year, GBD – Global Burden of Disease Study.

**Figure 5:**
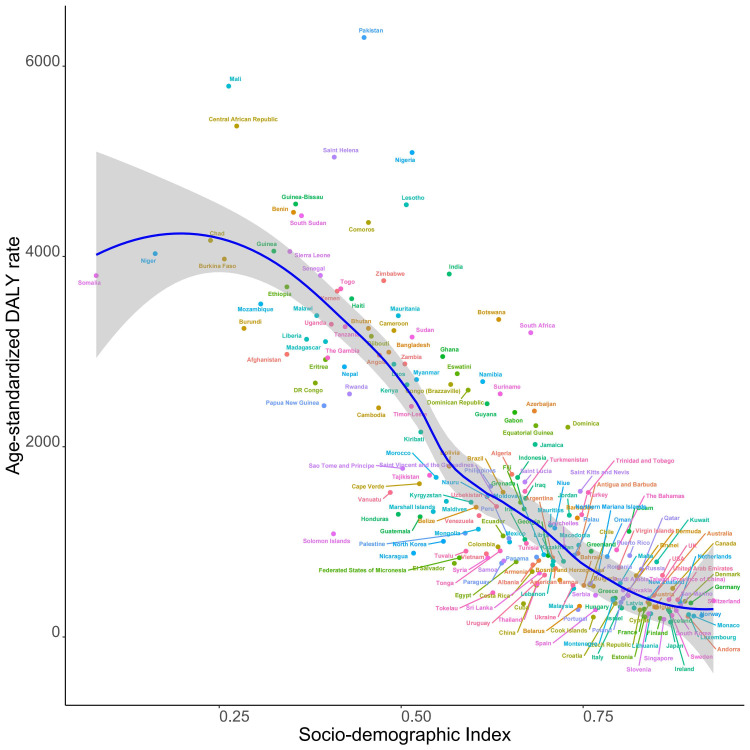
Age-standardised DALY rates attributable to preterm birth and low birth weight across 204 countries and territories by socio-demographic index in 2019. DALY – disability-adjusted life year.

There has been a global decline in the ASR of DALYs linked with PBLBW, with the reduction being particularly pronounced in low SDI regions. Sub-Saharan Africa showed a pattern of initial increase followed by a decrease, reaching its peak when the SDI was around 0.6 (**Figure 4**). The trend indicates that with the rise in SDI (starting from approximately 0.2), there is a corresponding decrease in the age-standardised DALY rates until an SDI of about 0.9 is reached; beyond this point, the rates tend to plateau with further increases. When considering only SDI, the age-standardised DALY ratios for Kiribati, Mali, and the Central African Republic were much higher than those of other nations (**Figure 5**).

## DISCUSSION

We utilised data from the GBD Study 2019 to comprehensively analyse the worldwide distribution, temporal trends, regional variances, and the association with socioeconomic factors in deaths and DALYs attributed to PBLBW. Our findings reveal that between 1990–2019, there was a significant global decrease in PBLBW-related deaths and DALYs, with reductions of 42.2% and 40.5%, respectively. Throughout this period, all 21 regions defined by the GBD reported declines in mortality and DALY rates associated with PBLBW. Among the 204 countries analysed, the Cook Islands demonstrated the most substantial decrease in mortality rates and DALYs, while Guam experienced the largest increase. In terms of SDI regions, from 1990 to 2019, middle and high SDI regions witnessed significant reductions in both age-standardised death and DALY rates for PBLBW. In contrast, low SDI regions recorded an average annual decrease of 3.2% in age-standardised death rates but faced an average annual increase of 6.4% in age-standardised DALY rates. Furthermore, our attribution analysis identified the neonatal and infancy periods as critical intervals for PBLBW-related mortality. Neonatal disorders emerged as the leading cause, with LRIs and SIDS also presenting as substantial contributing factors. This nuanced examination underscores the complex interplay between socioeconomic factors, regional disparities, and health outcomes in the context of PBLBW.

The occurrence of PBLBW infants is multifactorial, involving maternal health, lifestyle in modern society, inequities in health care resources, nutritional status, and environmental conditions [24]. A previous study showed that the global age-standardised incidence rate for PTB has decreased marginally by an average of 0.19% per year over the last three decades, with some regions even seeing an uptick [4]. However, there was a significant downward trend in global PBLBW-related deaths, DALYs and ASR for both females and males from 1990 to 2019 observed in our study, which can be attributed to medical advancements, including development in medical equipment, respiratory support, and nutritional management [25]. The administration of repeat doses of prenatal corticosteroids for women at risk of PTB and surfactant replacement therapy during the early postnatal period, for instance, has improved respiratory outcomes and reduced mortality rates in premature infants [26,27]. In addition, key preventative measures such as enhanced maternal health care services, healthy diet and nutrition promotion, high-quality prenatal care, and education for pregnant women have played a critical role [28]. However, our study reveals a startling trend. Guam has witnessed the most significant surge in the incidence of PTB over the past three decades. This phenomenon is particularly noteworthy given the unique health profile of Guam and other Pacific Islanders, who are grappling with high incidences of obesity and associated non-communicable diseases. These health issues pose a considerable threat, increasing the likelihood of adverse pregnancy outcomes such as preterm or extremely preterm births [29]. Additionally, the disparities in residency status and access to American social services among certain Pacific Islander communities present formidable social, economic, and political challenges. These challenges exacerbate the risks faced by pregnant women and their infants, further compounding the issue of preterm births in these communities [30].

Premature infants who are born with a shorter gestational age and lower birth weight are immediately confronted with severe hypoxic respiratory failure due to immature alveolar development, necessitating the administration of surfactants and the utilisation of ventilators [31]. Furthermore, PBLBW infants need to complete the transition from foetal circulation to neonatal circulation in the first week of life, which can easily lead to complications such as intraventricular haemorrhage (IVH) due to respiratory and hemodynamic fluctuations, resulting in death or serious neurological sequelae. From a global perspective, three-quarters of newborn deaths occur in the first week after birth, especially in PBLBW infants in middle and low-income countries [32]. Therefore, conducting age-stratified research on deaths and DALYs associated with PBLBW holds significant importance. Our study reveals that the age-specific rates of PBLBW-related deaths and DALYs decrease progressively as age advances. Remarkably, the zero to six days age group demonstrates the highest incidence of deaths and DALYs, along with the highest PBLBW-related death and DALY rates, which aligns with the outcomes of previous investigations. Stakeholders should identify and emphasise preventable and treatable predictive factors as early as possible. For example, receiving delayed umbilical cord clamping and umbilical cord milking during delivery, as well as early hemodynamic screening and physiological guided care, can be avenues to further reduce the risk of IVH within one week after birth and improve neural outcomes in premature infants [33,34].

Additionally, significant variations were observed in the age-standardised death and DALYs rates for PBLBW across different regions and countries. It was noted that from 1990 to 2019, the decline in age-standardised death and DALYs rates for PBLBW in low-SDI areas lagged considerably behind those of middle- and high-SDI regions and the global average. Previous studies firmly established that sociodemographic factors stemming from limited maternal education and low family wealth determine unfavourable birth outcomes, encompassing PTB and LBW [35]. Notably, socioeconomic disparities persist as the foremost predictor of adverse perinatal outcomes and a pivotal factor that significantly impacts the outcome of PBLBW [35]. For instance, COVID-19 mRNA vaccination during pregnancy seems to be associated with a reduction in stillbirths [36], while residing in urban areas and having a higher income level correlate with a higher vaccination rate [37]. Therefore, it is evident that although PBLBW is prevalent in high- and middle-income countries, the majority of child deaths resulting from PBLBW occur in low- and middle-income regions such as South Asia and sub-Saharan Africa [38]. In high-income nations, the vast majority of extremely preterm infants survive, and their prognosis is improved through continued intensive care, as well as research into both short and long-term complications. Conversely, in low-income countries, most preterm infants born after 32 weeks succumb to death or develop moderate to severe bronchopulmonary dysplasia or neurodevelopmental impairment due to the absence of even basic medical interventions, such as antenatal corticosteroid therapy, skilled birth attendance, breastfeeding support, and primary care for infections and respiratory distress [39]. Studies have identified that preterm births account for 49% and 40% of all neonatal deaths in South Asia and sub-Saharan Africa, respectively. A higher proportion of LBW and small for gestational age infants are found in these regions, possibly linked to poor maternal nutrition. Supplementation of micronutrients and protein-energy to pregnant women significantly reduces the incidence of small for gestational age births and LBW [40]. PTB remains a critical public health issue globally, with low-income countries particularly in need of robust interventions to reduce rates of prematurity and enhance survival and quality of life for preterm infants. There is also a necessity for stronger international collaboration, seeking support and resources from the global community.

During the pre-surfactant era, the majority of preterm neonates, especially those of extremely low gestational age, succumbed to immaturity and respiratory failure within days following birth. However, the advent of antenatal steroids, surfactant therapy, enhanced respiratory support technologies, and improvements in neonatal care have precipitated a shift in mortality causes from pulmonary aetiologies to non-pulmonary complications such as IVH, necrotising enterocolitis, nosocomial infections, and bronchopulmonary dysplasia [41]. Research indicates that mortality due to respiratory distress syndrome and severe IVH predominantly occurs early in life, with late-onset sepsis or necrotising enterocolitis potentially leading to deaths in subsequent weeks. In contrast, bronchopulmonary dysplasia emerges as a primary cause of death beyond two months of age [42]. Preterm infants, particularly those afflicted with bronchopulmonary dysplasia, are at an elevated risk for recurrent wheezing, asthma, and respiratory tract infections compared to their full-term counterparts, identifying them as high-risk individuals [43]. bronchopulmonary dysplasia, the most common complication among preterm infants, affects approximately 45% of babies born before 29 weeks of gestation [44], often manifesting as poor lung tissue development, heightened airway reactivity, and reduced lung function, consequently increasing susceptibility to both acute and chronic respiratory conditions. Moreover, LRIs not only pose a life-threatening risk but also significantly impact medical expenses and the quality of life for the families involved, especially in low- and middle-income countries [45]. This aligns with findings from our study wherein, for the year 2019, LRIs ranked as the second leading cause of age-standardised deaths and DALYs among infants under one year of age, subsequent only to neonatal disorders. Henceforth, it is imperative to identify preterm infants at heightened risk for LRIs hospitalisation during follow-up, as these patients stand to gain from more efficacious and focused preventive or treatment interventions, ultimately mitigating the substantial health care and clinical burden associated with LRIs.

SIDS, also known as ‘crib death,’ is a condition characterised by the unexpected demise of an infant, often during sleep. The syndrome’s onset cannot be predicted based on the health status or medical history of the child, and postmortem examinations typically do not reveal a definitive cause of death [46]. Annually, in the United States, around 3600 infants experience sudden death, with preterm and low birth weight infants facing a two to 3-fold increased risk of sudden unexpected death compared to their healthy full-term counterparts [47]. This risk is inverse to gestational age and birth weight, potentially associated with immature respiratory neural control mechanisms in preterm infants [48]. Our investigation indicates that SIDS remains a significant contributor to preterm infant mortality worldwide, exhibiting a nearly 10% annual increase in ASR as of 2019. The advent of the Back to Sleep campaign, aimed at preventing SIDS, precipitated a drastic reduction in its incidence across numerous nations. Current guidelines underscore that all infants should sleep in safe settings – on their backs, on firm surfaces, devoid of soft objects and loose bedding, without head coverings, protected from overheating, and co-rooming rather than co-bedding. Furthermore, avoiding prenatal and postnatal exposure to tobacco, alcohol, and illicit substances, coupled with advocacy for breastfeeding on demand and pacifier use during sleep, are measures recommended to safeguard against SIDS [49,50]. Nevertheless, ongoing research is essential to elucidate the precise mechanisms involved and to identify other viable preventive strategies. The widely accepted model for SIDS is the ‘triple-risk model,’ which assumes that the occurrence of SIDS results from the concurrent presence of vulnerable infants, critical developmental periods, and exogenous stressors [51]. Research has indicated that infants with SIDS exhibit a decrease in butyrylcholinesterase activity in the early postnatal period, which is associated with their autonomic cholinergic dysfunction and inherent fragility [52]. Additionally, subclinical infections and inflammation may play pivotal roles in the pathogenesis of SIDS [53]. These discoveries represent the potential to identify infants at risk for SIDS before their demise, serving as potential biomarkers and paving new avenues for future research on specific intervention measures.

Our study reveals profound gender disparities in the global burden of PBLBW. The underlying reasons for these disparities remain elusive. Previous retrospective cohort studies have demonstrated a gender gap in the mortality rates of PBLBW neonates, with males often exhibiting poorer overall survival rates and greater risks of morbidity, highlighting a so-called ‘male disadvantage’ [54]. Stark et al. observed that male neonates born between 24-28 weeks of gestation exhibited increased microvascular blood flow, which could contribute to lower systemic blood flow and hypotension during the critical first 24 hours of life [55]. Nevertheless, the intricate physiological mechanisms underlying the male disadvantage remain an area of ongoing research. Addressing these gender differences in DALYs associated with PBLBW is imperative and requires the urgent design and implementation of targeted strategies. Current policies and plans often overlook a gender-based perspective despite the need for a more nuanced approach in addressing the specific health challenges faced by males and females affected by PBLBW.

After 2019, remarkable advancements have been achieved in neonatal care and public health policies, exerting a profound positive influence on the trends related to PBLBW, even if such data were unavailable for this analysis. Implementing cutting-edge respiratory support techniques and meticulous nutritional support strategies has significantly reduced mortality rates and the risks associated with various complications [56–58]. Additionally, government-led health education initiatives have effectively raised awareness among pregnant women regarding prenatal care by disseminating nutritional knowledge and providing health consultations. This, in turn, has led to a decrease in the incidence of PBLBW infants. Furthermore, the government has intensified its medical support for impoverished regions and disadvantaged populations, ensuring equitable and high-quality health care services for all [59].

Undoubtedly, it is important to acknowledge several limitations in this study. First, variations in health systems, data recording standards, and resource allocations across nations and regions may result in incomplete or unavailable data. Such missingness can lead to inaccurate estimations of the burden of preterm birth diseases in specific regions or populations, ultimately affecting the reliability of our research conclusions. Second, reporting bias may arise due to disparities in diagnostic criteria, inconsistencies in medical practices, and differing reporting habits among patients and doctors. Furthermore, the significant variations in economic levels, cultural norms, medical resources, and policy environments across countries can significantly impact the incidence, mortality, and overall disease burden of PBLBW. Lastly, while the GBD 2019 database serves as our primary data source, the need for triangulation with other data sources remains paramount to enhance the reliability and validity of our results. However, due to constraints in time and resources, we may be unable to conduct such validations across all countries, which represents a potential limitation in our study.

## CONCLUSIONS

PBLBW has significantly contributed to the global burden of disease, influencing both mortality rates and DALYs, particularly among neonates under 28 days and in regions with low SDI. To effectively address this issue, deeper insights are needed into the causes, risk factors, and preventative measures for PBLBW. Policymakers worldwide must prioritise PBLBW on their agendas and allocate appropriate resources to enhance the health outcomes for these infants. This includes emphasising the potential of direct interventions, such as prenatal care, micronutrient supplementation, early breastfeeding, kangaroo mother care, and bubble continuous positive airway pressure, which, when delivered as part of a whole-system strengthening approach, can significantly reduce preterm birth and neonatal mortality in low- and middle-income settings. Ultimately, these efforts aim to enhance the overall survival rates and quality of life for infants affected by PBLBW, thus contributing to achieving sustainable development goals.

## Additional material


Online Supplementary Document
