# Supplementary material for: The global burden of disease attributable to preterm birth and low birth weight in 204 countries and territories from 1990 to 2019: An analysis of the Global Burden of Disease Study
Source: J Glob Health. 2024 Jul 12;14:04109. doi: 10.7189/jogh.14.04109 (PMC11239190; doi:10.7189/jogh.14.04109)
Supplement: Online Supplementary Document [file jogh-14-04109-s001.pdf]

Table S1. Deaths and DALYs attributable to PBLBW in 2019, by sex, global and SDI regions.

Region	Sex	Deaths		DALYs	
		1990 count, n (95% UI)	2019 count, n (95% UI)	1990 count, n (95% UI)	2019 count, n (95% UI)
Global	Both	3167605.3 (2947281.5, 3400387.1)	1805481.5 (1537291.4, 2143499.8)	286456376.9 (266580199.0, 307079118.1)	170087071.4 (145912387.6, 201254338.7)
Global	Female	1372344.0 (1271510.2, 1479928.5)	773916.1 (662881.0, 908535.9)	124415624.4 (115437143.7, 134216821.5)	73526290.8 (63565730.3, 85760680.8)
Global	Male	1795261.3 (1661473.4, 1938969.9)	1031565.4 (868896.3, 1237696.3)	162040752.5 (149869531.5, 174572880.0)	96560780.6 (82119146.8, 114619448.4)
High SDI	Both	45509.1 (42914.9, 48106.0)	18263.0 (16285.0, 20377.0)	4948586.7 (4643001.6, 5294732.5)	2737556.7 (2452725.6, 3065760.7)
High SDI	Female	19407.8 (18103.2, 20751.7)	8027.3 (7140.6, 8885.9)	2192573.1 (2037668.1, 2350090.9)	1278797.8 (1137650.6, 1435585.3)
High SDI	Male	26101.3 (24479.3, 27858.5)	10235.7 (8941.9, 11585.2)	2756013.6 (2579782.9, 2953368.5)	1458758.9 (1299238.8, 1638661.2)
High-middle SDI	Both	227072.8 (208186.9, 247549.2)	53986.8 (46288.9, 63214.4)	21225099.1 (19452998.1, 23022456.1)	6298908.5 (5528700.3, 7196592.7)
High-middle SDI	Female	95541.5 (87636.0, 103940.5)	23483.7 (20308.0, 27168.5)	9031953.4 (8308743.7, 9775659.5)	2846524.2 (2514570.2, 3221172.9)
High-middle SDI	Male	131531.3 (120192.8, 144464.6)	30503.1 (25887.5, 35995.2)	12193145.7 (11152747.0, 13351444.8)	3452384.3 (3005445.3, 3990682.6)
Middle SDI	Both	737513.0 (686411.3, 791087.6)	263975.1 (223367.6, 313290.1)	66998521.1 (62419492.5, 71740180.6)	26326859.4 (22604751.1, 30766650.6)
Middle SDI	Female	312660.9 (290567.3, 335384.2)	112890.4 (96469.6, 133237.6)	28506107.9 (26538069.9, 30534983.1)	11458782.8 (9930136.9, 13327042.3)
Middle SDI	Male	424852.1 (391412.4, 458785.3)	151084.6 (126529.2, 181098.2)	38492413.1 (35471984.2, 41603434.0)	14868076.6 (12674633.9, 17535205.5)
Low-middle SDI	Both	1323975.3 (1227213.7, 1426709.0)	650439.6 (552195.4, 762259.8)	118820390.7 (110181689.1, 128020171.1)	60510322.1 (51863389.9, 70809345.0)
Low-middle SDI	Female	586010.5 (532326.3, 641837.4)	287287.2 (246073.8, 334687.1)	52620570.5 (47870992.8, 57582098.3)	26831068.4 (23184729.4, 31070670.3)
Low-middle SDI	Male	737964.8 (679260.5, 800031.1)	363152.3 (304790.5, 431333.2)	66199820.2 (60938950.5, 71703122.0)	33679253.7 (28438142.0, 39788406.8)
Low SDI	Both	832131.3 (762959.5, 904051.8)	817801.1 (673798.3, 997438.8)	74335850.4 (68172272.4, 80714140.9)	74118252.1 (61217267.0, 89966174.6)
Low SDI	Female	358134.5 (327871.9, 389683.9)	341800.9 (285529.0, 408996.7)	32010484.6 (29296104.5, 34816738.0)	31070775.7 (26089234.9, 37063106.0)
Low SDI	Male	473996.8 (432275.8, 518808.4)	476000.2 (387105.1, 587432.7)	42325365.9 (38615200.7, 46282157.5)	43047476.4 (35162853.6, 52912045.8)

DALY= disability-adjusted life year; PBLBW= preterm birth and low birth weight; SDI= Socio-demographic Index.

Table S2. Age standardized Deaths and DALYs attributable to PBLBW in 2019 and percentage change from 1990 to 2019, by GBD regions.

Region	Deaths			DALYs		
	1990 ASR*, n (95% UI)	2019 ASR*, n (95% UI)	Percentage change in ASR (1990–2019), % (95% CI)	1990 ASR*, n (95% UI)	2019 ASR*, n (95% UI)	Percentage change in ASR (1990–2019), % (95% CI)
Andean Latin America	36.5 (33.1, 40.3)	12.8 (9.6, 16.8)	-64.9% (-74.8, -53.3)	1230.7 (942.2, 1579.1)	3328.2 (3024.9, 3658.0)	-63.0% (-72.6, -51.7)
Australasia	6.5 (6.0, 7.0)	2.8 (2.3, 3.4)	-56.2% (-64.7, -46.3)	372.0 (320.5, 428.9)	707.1 (656.1, 771.4)	-47.4% (-55.1, -38.9)
Caribbean	35.0 (30.6, 39.2)	27.4 (20.9, 34.9)	-21.6% (-42.6, 3.9)	2555.8 (1967.4, 3208.2)	3236.4 (2852.8, 3621.0)	-21.0% (-41.1, 3.5)
Central Asia	27.6 (25.2, 30.1)	14.4 (11.9, 17.5)	-47.9% (-56.8, -36.4)	1364.0 (1144.1, 1641.5)	2528.0 (2319.0, 2755.5)	-46.0% (-54.8, -34.8%)
Central Europe	15.0 (14.3, 15.8)	3.7 (2.9, 4.7)	-75.2% (-80.8, -68.8)	439.3 (358.9, 524.9)	1438.8 (1373.2, 1517.9)	-69.5% (-75.0, -63.4)
Central Latin America	28.9 (25.2, 32.8)	10.6 (8.2, 13.3)	-63.3% (-72.4, -52.7)	1043.0 (826.7, 1282.6)	2652.3 (2332.9, 2986.0)	-60.7% (-69.6, -50.1)
Central Sub-Saharan Africa	54.3 (45.2, 63.6)	31.4 (26, 37.9)	-42.2% (-53.1, -28.6)	2855.2 (2376.6, 3425.5)	4858.9 (4044.7, 5684.8)	-41.2% (-52.1, -27.6)
East Asia	23.2 (20.6, 26.1)	5.3 (4.5, 6.1)	-77.4% (-81.1, -72.7)	552.0 (486.3, 628.2)	2119.4 (1883.3, 2373.7)	-74.0% (-77.8, -69.3)
Eastern Europe	13.5 (12.6, 15.1)	3.8 (3.0, 4.6)	-72.3% (-78.2, -66.0)	427.7 (361.3, 499.1)	1286.4 (1193.2, 1426.7)	-66.8% (-72.7, -60.7)
Eastern Sub-Saharan Africa	61.7 (55.6, 67.9)	36.6 (29.1, 46.1)	-40.6% (-51.8, -26.1)	3355.4 (2686.2, 4199.3)	5530.1 (4998.8, 6080.4)	-39.3% (-50.4, -24.7)
High-income Asia Pacific	4.4 (4.0, 5.1)	1.3 (1.1, 1.4)	-71.0% (-75.8, -66.1)	196.1 (173.3, 221.0)	468.5 (423.3, 530.5)	-58.1% (-63.6, -52.7)
High-income North America	8.7 (8.3, 9.2)	5.4 (4.9, 5.9)	-38.1% (-44.6, -31.3)	635.3 (577.4, 696)	921.8 (870.3, 982.5)	-31.1% (-36.8, -25.1)
North Africa and Middle East	52.8 (46.4, 59.0)	18.0 (15.3, 21.1)	-66.0% (-71.5, -59.6)	1740.7 (1499.9, 2042.6)	4808.8 (4242.8, 5357.5)	-63.8% (-69.4, -57.2)
Oceania	29.5 (24.1, 35.2)	24.3 (18.0, 32.7)	-17.5% (-37.5, 7.8)	2237.2 (1667.9, 2975.7)	2698.3 (2219.9, 3210.5)	-17.1% (-36.5, 7.6)
South Asia	81.9 (74.9, 89.5)	45.4 (38.4, 53.7)	-44.6% (-54.1, -33.0)	4229.2 (3603.1, 4965.3)	7388.4 (6764.9, 8071.0)	-42.8% (-52.1, -31.0)
Southeast Asia	39.0 (35.3, 43.2)	16.5 (13.3, 20.2)	-57.8% (-66.4, -47.5)	1554.1 (1262.6, 1891.6)	3543.9 (3202.9, 3916.7)	-56.1% (-64.6, -46.1)
Southern Latin America	20.0 (19.1, 20.9)	7.4 (5.7, 9.4)	-63.0% (-71.8, -52.6)	758.9 (604.6, 938.3)	1876.2 (1785.2, 1960.0)	-59.6% (-68.0, -49.4)
Southern Sub-Saharan Africa	42.6 (36.5, 49.6)	35.8 (28.1, 46.1)	-15.9% (-35.2, 10.7)	3363.4 (2692.8, 4294.8)	3896.1 (3358.9, 4519.4)	-13.7% (-32.7, 12.1)
Tropical Latin America	43.3 (37.9, 50.0)	15.2 (12.0, 18.8)	-64.8% (-73.5, -55.1)	1492.1 (1211.3, 1804.0)	3919.2 (3429.7, 4514.6)	-61.9% (-70.9, -52.0)
Western Europe	6.3 (6.0, 6.7)	2.6 (2.2, 3.1)	-58.4% (-66.4, -50.2)	338.9 (292.9, 388.2)	661.7 (625.8, 707.7)	-48.8% (-56.2, -41.5)
Western Sub-Saharan Africa	72.3 (65.5, 78.7)	50.6 (42.0, 61.4)	-29.9% (-42.0, -14.9)	4567.1 (3796.8, 5527.1)	6460.4 (5861.2, 7027.3)	-29.3% (-41.3, -14.3)

DALY= disability-adjusted life year; PBLBW= preterm birth and low birth weight; GBD= global burden of disease; ASR= age standardized rate.

*Per 100 000 people.

S3 Table. Age standardized Deaths and DALYs attributable to PBLBW in 2019 and percentage change from 1990 to 2019, by countries.

Countries	Deaths			DALYs		
	1990 ASR*, n (95% UI)	2019 ASR*, n (95% UI)	Percentage change in ASR (1990–2019),% (95% CI)	1990 ASR*, n (95% UI)	2019 ASR*, n (95% UI)	Percentage change in ASR (1990–2019), % (95% CI)
Afghanistan	67.3 (53.0, 85.2)	32.2 (24.7, 40.5)	-52.2% (-61.7, -39.6)	6092.1 (4805.8, 7672.7)	2971.4 (2304.4, 3709.3)	-51.2% (-60.6, -38.8)
Albania	19.8 (16.7, 23.1)	7.3 (4.9, 11.1)	-63.0% (-76.2, -44.3)	1881.0 (1606.9, 2188.2)	758.3 (540.2, 1100.0)	-59.7% (-72.0, -42.5)
Algeria	41.7 (32.6, 52.6)	17.9 (13.8, 22.7)	-57.0% (-68.5, -41.5)	3795.5 (3000.3, 4749.2)	1710.1 (1344.0, 2143.6)	-54.9% (-66.2, -39.7)
American Samoa	11.8 (9.5, 14.4)	6.7 (4.8, 8.9)	-43.3% (-58.4, -25.6)	1178.0 (972.5, 1403.6)	719.8 (547.5, 913.4)	-38.9% (-52.8, -22.8)
Andorra	4.0 (3.1, 5.0)	1.4 (1.0, 1.8)	-66.0% (-76.7, -49.2)	449.3 (370.2, 549.6)	217.6 (176.8, 269.1)	-51.6% (-62.7, -36.9)
Angola	72.9 (59.3, 88.2)	32.5 (26.7, 38.4)	-55.4% (-65.7, -42.0)	6511.4 (5313.5, 7871.2)	2962.9 (2452.8, 3486.6)	-54.5% (-65.1, -40.9)
Antigua and Barbuda	14.5 (12.0, 17.5)	8.4(6.0, 11.6)	-41.7% (-61.4, -16.6)	1420.5 (1186.9, 1688.4)	878.2 (651.1, 1165.9)	-38.2% (-56.6, -15.1)
Argentina	23.8 (22.6, 25.1)	8.4 (6.5, 10.5)	-64.9% (-72.9, -55.3)	2206.9 (2093.5, 2321.2)	833.3 (667.4, 1025.0)	-62.2% (-70.0, -53.0)
Armenia	31.3 (26.3, 36.7)	7.8 (5.9, 10.1)	-75.0% (-82.1, -65.8)	2879.6 (2441.4, 3372.5)	804.3 (638.6, 1017.3)	-72.1% (-79.2, -62.5)
Australia	6.5 (6.0, 7.1)	2.8 (2.3, 3.3)	-57.2% (-65.2, -48.0)	713.6 (656.0, 782.5)	366.1 (315.9, 420.1)	-48.7% (-56.0, -40.9)
Austria	6.0 (5.4, 6.8)	2.3 (1.9, 2.7)	-62.1% (-69.9, -53.7)	644.0 (580.4, 717.9)	313.6 (266.6, 361.4)	-51.3% (-59.3, -42.9)
Azerbaijan	44.5 (37.1, 53.7)	25.6 (20.6, 31.2)	-42.5% (-55.9, -24.6)	4033.8 (3364.0, 4859.8)	2375.5 (1920.3, 2880.4)	-41.1% (-54.4, -23.5)
Bahamas	18.1 (14.9, 21.5)	8.9 (6.6, 11.6)	-50.7% (-64.8, -30.4)	1734.2 (1458.4, 2030.6)	915.4 (710.9, 1171.2)	-47.2% (-60.7, -28.7)
Bahrain	19.5 (16.5, 23.1)	4.2 (3.3, 5.4)	-78.6% (-84.2, -71.0)	1880.3 (1607.7, 2208.8)	542.5 (451.2, 661.5)	-71.1% (-77.5, -63.3)
Bangladesh	87.7 (77.6, 98.8)	31.5 (23.7, 40.3)	-64.1% (-73.7, -52.7)	7989.9 (7091.6, 8957.7)	2994.0 (2302.0, 3785.2)	-62.5% (-72.3, -51.2)
Barbados	19.1 (15.9, 22.6)	12.5 (8.6, 17.8)	-34.5% (-57.0, -2.9)	1845.6 (1560.5, 2148.2)	1250.9 (900.4, 1721.4)	-32.2% (-53.7, -3.6)
Belarus	10.0 (8.3, 12.0)	2.5 (1.7, 3.3)	-74.8% (-84.3, -65.1)	992.0 (833.9, 1169.6)	323.1 (250.8, 398.9)	-67.4% (-76.3, -57.0)
Belgium	6.1 (5.6, 7.0)	2.3 (1.9, 2.8)	-61.9% (-70.6, -52.3)	642.5 (585.5, 719.5)	312.2 (262.1, 366.9)	-51.4% (-59.9, -42.2)
Belize	28.9 (25.2, 32.9)	13.9 (11.2, 16.8)	-52.1% (-63.2, -39.2)	2698.4 (2367.5, 3056.7)	1364.1 (1129.2, 1629.2)	-49.4% (-60.6, -37.0)
Benin	72.5 (58.3, 89.2)	49.6 (40.0, 61.8)	-31.6% (-49.0, -9.3%)	6483.5 (5212.3, 7978.4)	4463.7 (3619.0, 5550.1)	-31.2% (-48.5, -9.0)
Bermuda	8.9 (7.7, 10.1)	3.9 (2.7, 5.6)	-55.7% (-71.0, -35.4)	942.6 (830.0, 1056.6)	494.5 (380.8, 644.5)	-47.5% (-60.9, -30.1)

Bhutan	93.6 (75.4, 113.8)	34.4 (24.6, 47.2)	-63.2% (-74.6, -48.6)	8495.5 (6884.0, 10274.1)	3244.5 (2371.8, 4360.3)	-61.8% (-73.0, -47.2)
Bolivia (Plurinational State of)	49.6 (43.7, 56.4)	19.4 (15.0, 24.4)	-60.9% (-70.6, -49.1)	4463.1 (3937.1, 5056.3)	1795.0 (1405.8, 2242.1)	-59.8% (-69.5, -48.0)
Bosnia and Herzegovina	19.6 (16.9, 22.6)	5.1 (4.1, 6.3)	-73.8% (-80.1, -67.0)	1909.4 (1663.0, 2184.7)	597.3 (501.6, 701.6)	-68.7% (-75.1, -61.9)
Botswana	42.4 (34.4, 50.3)	35.4 (25.7, 49.4)	-16.6% (-39.8, 13.7)	3936.7 (3229.1, 4635.0)	3337.7 (2476.8, 4622.6)	-15.2% (-37.3, 13.7)
Brazil	44.0 (38.4, 50.9)	15.6 (12.3, 19.2)	-64.6% (-73.3, -54.7)	3978.4 (3477.1, 4599.4)	1521.3 (1237.0, 1842.6)	-61.8% (-70.8, -51.7)
Brunei Darussalam	6.4 (5.6, 7.4)	6.2 (4.7, 8.3)	-2.7% (-28.3, 30.5)	652.5 (573.4, 736.1)	644.3 (506.8, 829.3)	-1.3% (-23.9, 28.1)
Bulgaria	10.5 (9.2, 12.3)	5.0 (3.8, 6.4)	-52.4% (-64.5, -37.0)	1025.6 (915.7, 1188.0)	533.7 (427.6, 660.8)	-48.0% (-59.3, -33.4)
Burkina Faso	69.8 (59.3, 80.4)	44.2 (34.2, 57.2)	-36.7% (-52.0, -17.1)	6235.3 (5310.1, 7176.5)	3973.0 (3082.1, 5129.3)	-36.3% (-51.5, -16.9)
Burundi	60.7 (53.3, 68.6)	35.8 (29.8, 43.2)	-41.1% (-52.2, -28.1)	5429.5 (4760.6, 6135.3)	3243.8 (2710.4, 3896.6)	-40.3% (-51.3, -27.4)
Cabo Verde	33.3 (29.8, 36.6)	16.3 (11.7, 22.2)	-50.9% (-65.7, -32.1)	3078.9 (2767.7, 3380.1)	1610.8 (1188.6, 2131.2)	-47.7%(-62.1, -29.5)
Cambodia	62.7 (53.1, 72.8)	26.0 (19.7, 34.8)	-58.5% (-69.4, -42.8)	5681.7 (4827.2, 6556.4)	2408.5 (1848.0, 3182.2)	-57.6% (-68.3, -42.4)
Cameroon	54.0 (44.2, 64.1)	35.6 (27.7, 44.2)	-34.0%(-50.9, -11.3)	4833.0 (3962.0, 5733.4)	3221.5 (2517.2, 3979.4)	-33.3% (-50.1, -10.8)
Canada	5.8 (5.3, 6.5)	4.3 (3.8, 4.9)	-25.3% (-37.9, -12.0)	632.2 (577.8, 698.9)	510.5 (447.3, 575.7)	-19.3% (-30.7, -7.5)
Central African Republic	76.4 (65.1, 89.3)	60.0 (46.6, 76.9)	-21.4% (-40.6, 2.6)	6821.9 (5808.2, 7964.8)	5370.9 (4183.9, 6867.7)	-21.3% (-40.3, 2.6)
Chad	64.8 (54.8, 75.4)	46.5 (37.5, 57.6)	-28.2% (-43.2, -8.5)	5781.9 (4897.7, 6724.0)	4168.6 (3362.2, 5158.5)	-27.9% (-42.8, -8.3)
Chile	11.4 (10.7, 12.2)	4.8 (3.5, 6.4)	-57.8% (-69.4, -43.6)	1118.7 (1045.3, 1192.3)	548.9 (422.8, 699.9)	-50.9% (-61.7, -36.9)
China	23.1 (20.5, 25.9)	5.2 (4.4, 6.0)	-77.6% (-81.5, -72.9)	2107.8 (1882.0, 2361.0)	543.4 (478.7, 621.3)	-74.2% (-78.2, -69.6)
Colombia	25.2 (22.0, 28.8)	9.4 (6.2, 13.4)	-62.6% (-75.8, -45.1)	2333.4 (2049.8, 2645.7)	947.4 (664.1, 1295.6)	-59.4% (-72.3, -42.4)
Comoros	83.5 (72.4, 94.9)	47.3 (37.1, 59.7)	-43.3% (-57.0, -27.5)	7520.9 (6516.3, 8529.8)	4357.1 (3453.5, 5463.8)	-42.1% (-55.3, -26.3)
Congo	44.2 (37.8, 51.1)	28.6 (23.7, 34.1)	-35.3% (-47.8, -19.3)	3993.2 (3421.7, 4606.2)	2654.4 (2215.3, 3149.2)	-33.5% (-45.9, -18.1)
Cook Islands	13.6 (10.5, 17.6)	1.0 (0.6, 1.4)	-93.0% (-95.6, -90.0)	1334.2 (1051.1, 1689.0)	208.8 (162.6, 259.4)	-84.4% (-88.2, -80.1)
Costa Rica	12.9 (10.8, 15.1)	6.5 (4.5, 8.9)	-49.7% (-65.0, -28.8)	1261.3 (1073.5, 1460.1)	687.3 (504.6, 903.2)	-45.5% (-59.8, -26.3)
Croatia	9.7 (8.8, 10.5)	2.8 (2.1, 3.8)	-71.1% (-79.1, -60.7)	967.8 (883.0, 1047.3)	353.5 (278.3, 444.0)	-63.5% (-71.3, -53.7)
Cuba	8.8 (8.1, 9.5)	2.8 (1.9, 3.6)	-68.0% (-77.6, -58.2)	901.7 (831.1, 973.1)	350.1 (273.4, 422.2)	-61.2% (-69.6, -52.5)
Cyprus	13.6 (11.8, 15.6)	2.8 (2.0, 3.8)	-79.3% (-85.3, -71.5)	1307.1 (1145.9, 1488.7)	346.7 (276.4, 437.7)	-73.5% (-79.5, -65.5)
Czechia	10.0 (9.3, 11.1)	2.0 (1.5, 2.6)	-80.0% (-86.5, -73.1)	994.4 (923.1, 1097.2)	284.2 (231.1, 345.1)	-71.4% (-77.9, -64.9)

Côte d'Ivoire	72.2 (59.4, 84.6)	55.9 (44.9, 68.0)	-22.6% (-41.0, 1.1)	6465.3 (5337.4, 7561.9)	5044.1 (4060.1, 6128.0)	-22.0% (-40.4, 1.6)
Democratic People's Republic of Korea	36.5 (28.0, 46.1)	10.3 (7.9, 13.3)	-71.9% (-77.8, -63.5)	3342.5 (2589.7, 4193.2)	1005.1 (799.5, 1282.0)	-69.9% (-76.1, -62.0)
Democratic Republic of the Congo	48.4 (39.7, 57.8)	29.4 (23.4, 36.5)	-39.4% (-52.6, -23.2)	4330.9 (3549.4, 5162.5)	2669.8 (2131.8, 3308.3)	-38.4% (-51.6, -22.2)
Denmark	5.2 (4.6, 5.8)	3.2 (2.4, 4.0)	-38.7% (-54.8, -18.9)	551.0 (496.0, 611.2)	375.3 (300.1, 458.5)	-31.9% (-46.5, -14.6)
Djibouti	51.4 (43.4, 59.7)	34.3 (27.0, 43.4)	-33.4% (-49.5, -13.7)	4643.7 (3927.5, 5385.4)	3161.2 (2506.6, 3992.4)	-31.9% (-47.8, -12.6)
Dominica	18.4 (15.1, 22.1)	23.3 (16.3, 32.8)	26.7% (-13.1, 83.4)	1766.3 (1465.7, 2096.2)	2205.0 (1578.7, 3041.0)	24.8% (-12.5, 77.3)
Dominican Republic	42.6 (35.8, 49.8)	27.6 (19.3, 37.6)	-35.3% (-55.5, -6.7)	3919.0 (3317.3, 4556.4)	2595.1 (1859.6, 3507.3)	-33.8% (-53.5, -6.0)
Ecuador	26.0 (21.8, 30.8)	11.0 (7.5, 15.4)	-57.8% (-72.0, -38.0)	2397.0 (2026.2, 2825.3)	1062.4 (757.6, 1468.9)	-55.7% (-69.4, -36.8)
Egypt	46.6 (38.5, 55.1)	6.9 (4.2, 10.3)	-85.1% (-91.1, -76.9)	4279.6 (3560.6, 5054.6)	790.4 (536.7, 1090.5)	-81.5% (-87.7, -73.2)
El Salvador	31.6 (25.6, 38.1)	7.6 (5.1, 10.8)	-76.1% (-84.9, -63.2)	2908.5 (2384.7, 3487.7)	774.0 (553.5, 1060.2)	-73.4% (-82.3, -61.0)
Equatorial Guinea	64.3 (53.3, 76.5)	23.8 (15.5, 34.0)	-63.1% (-75.5, -47.5)	5766.7 (4773.4, 6848.0)	2221.2 (1489.9, 3136.8)	-61.5% (-73.7, -46.2)
Eritrea	57.2 (48.9, 66.4)	31.5 (22.8, 43.5)	-45.0% (-61.4, -23.8)	5166.0 (4427.4, 5986.6)	2915.6 (2161.0, 3977.3)	-43.6% (-59.8, -22.7)
Estonia	10.0 (8.9, 10.9)	1.4 (0.9, 1.7)	-86.5% (-91.6, -82.7)	999.5 (900.7, 1088.8)	211.3 (164.9, 256.5)	-78.9% (-83.5, -74.4)
Eswatini	38.3 (32.2, 45.1)	29.9 (24.1, 37.4)	-22.1% (-39.4, 0.5)	3492.6 (2941.2, 4094.2)	2767.3 (2261.4, 3442.4)	-20.8% (-37.6, 1.3)
Ethiopia	79.6 (69.9, 89.9)	40.1 (31.5, 51.2)	-49.7% (-60.7, -34.7)	7098.1 (6242.5, 8013.3)	3680.2 (2919.2, 4678.2)	-48.2% (-59.2, -33.3)
Fiji	17.6 (14.5, 21.3)	14.7 (10.6, 20.2)	-16.5% (-42.3, 20.1)	1668.8 (1387.1, 1999.6)	1415.6 (1037.0, 1892.6)	-15.2% (-39.7, 19.4)
Finland	4.3 (3.8, 4.8)	1.3 (0.9, 1.6)	-71.0% (-79.2, -62.6)	476.6 (429.9, 526.5)	195.6 (159.0, 230.6)	-59.0% (-66.6, -51.2)
France	4.4 (3.8, 5.0)	2.4 (2.0, 2.8)	-45.2% (-56.9, -31.4)	466.2 (412.6, 524.6)	293.1 (248.4, 339.6)	-37.1% (-48.1, -24.2)
Gabon	44.4 (37.6, 52.2)	24.7 (17.6, 34.6)	-44.4% (-60.7, -21.5)	4058.4 (3443.4, 4748.2)	2360.7 (1716.5, 3240.0)	-41.8% (-57.7, -19.4)
Gambia	59.4 (49.0, 71.1)	31.5 (23.0, 43.4)	-46.9% (-63.0, -24.8)	5363.1 (4431.8, 6396.6)	2934.4 (2167.3, 3976.8)	-45.3% (-61.3, -23.6)
Georgia	30.0 (25.8, 35.4)	8.4 (6.5, 10.7)	-72.0% (-79.2, -62.2)	2766.0 (2385.7, 3237.7)	856.92(677.52 to 1060.29)	-69.0% (-76.1, -59.5)
Germany	5.6 (4.9, 6.3)	2.6 (2.2, 2.9)	-53.9% (-62.1, -44.3)	622.6 (550.8, 695.7)	357.6 (314.5, 403.3)	-42.6% (-50.4, -33.6)
Ghana	59.4 (50.9, 68.7)	32.3 (22.6, 44.4)	-45.7% (-63.0, -22.3)	5342.7 (4590.1, 6160.2)	2947.8 (2080.2, 4016.0)	-44.8% (-62.0, -21.6)
Greece	9.2 (8.0, 11.2)	3.1 (2.5, 3.8)	-66.4% (-73.9, -56.2)	910.3 (801.9, 1091.6)	407.7 (341.0, 483.2)	-55.2% (-63.7, -44.7)
Greenland	31.6 (27.1, 36.8)	8.7 (5.6, 13.1)	-72.4% (-82.6, -57.5)	2927.7 (2516.7, 3387.3)	900.9 (613.4, 1290.4)	-69.2% (-79.3, -54.7)
Grenada	21.7 (16.9, 27.4)	13.7 (9.5, 19.3)	-36.8% (-58.7, -4.2)	2060.0 (1634.1, 2552.6)	1346.1 (976.6, 1831.2)	-34.7% (-55.5, -3.3)

Guam	8.4 (6.8, 10.1)	11.0 (8.4, 14.0)	30.7% (-4.1, 73.6)	865.9 (718.3, 1024.0)	1110.0 (876.5, 1383.6)	28.2% (-2.3, 64.6)
Guatemala	47.1 (41.9, 53.1)	13.0 (9.2, 17.7)	-72.4% (-80.4, -61.1)	4288.3 (3823.3, 4830.0)	1263.3 (921.9, 1688.3)	-70.5% (-78.5, -59.3)
Guinea	84.7 (74.5, 96.6)	45.1 (35.1, 56.4)	-46.7% (-59.0, -31.0)	7556.1 (6652.7, 8620.1)	4056.2 (3160.6, 5048.2)	-46.3% (-58.5, -30.7)
Guinea-Bissau	86.9 (69.7, 107.2)	50.3 (41.4, 59.7)	-42.2% (-52.9, -28.2)	7768.7 (6232.5, 9572.5)	4551.1 (3764.2, 5405.8)	-41.4% (-52.2, -27.5)
Guyana	56.5 (47.9, 66.2)	25.8 (18.0, 35.4)	-54.4% (-69.8, -34.5)	5207.3 (4433.3, 6099.4)	2451.7 (1757.6, 3307.9)	-52.9% (-68.0, -33.8)
Haiti	52.7 (39.4, 67.3)	39.0 (29.8, 49.4)	-26.0% (-48.9, 8.4)	4789.9 (3593.1, 6083.5)	3555.7 (2745.8, 4470.5)	-25.8% (-48.4, 7.9)
Honduras	32.3 (28.3, 36.8)	13.3 (10.1, 17.0)	-58.8% (-69.3, -45.1)	2982.6 (2623.6, 3386.3)	1289.6 (1003.7, 1615.7)	-56.8% (-67.2, -43.6)
Hungary	16.4 (14.5, 17.7)	3.0 (1.9, 4.0)	-82.1% (-88.2, -75.8)	1604.1 (1424.7, 1727.1)	402.6 (308.1, 500.5)	-74.9% (-80.8, -68.6)
Iceland	5.5 (4.7, 6.3)	1.8 (1.1, 2.9)	-66.3% (-80.2, -44.2)	588.9 (518.2, 673.8)	264.6 (194.9, 359.7)	-55.1% (-67.6, -36.9)
India	79.3 (70.6, 88.4)	40.6 (33.3, 50.5)	-48.7% (-59.5, -34.8)	7143.0 (6362.8, 7951.9)	3815.3 (3157.3, 4687.4)	-46.6% (-57.3, -32.9)
Indonesia	41.8 (36.4, 48.1)	17.9 (14.0, 22.5)	-57.1% (-67.7, -43.3)	3755.6 (3271.1, 4319.0)	1676.5 (1322.2, 2073.4)	-55.4% (-65.9, -41.7)
Iran (Islamic Republic of)	52.5 (43.0, 63.0)	10.0 (8.0, 12.3)	-81.0% (-86.1, -74.7)	4722.0 (3881.5, 5649.5)	1024.1 (842.6, 1240.0)	-78.3% (-83.6, -71.8)
Iraq	50.1 (42.8, 57.6)	14.8 (10.4, 20.5)	-70.4% (-78.9, -58.5)	4589.4 (3959.9, 5271.4)	1457.2 (1075.2, 1968.9)	-68.2% (-76.5, -56.5)
Ireland	5.5 (5.0, 6.2)	2.2 (1.7, 2.8)	-60.1% (-70.2, -47.3)	571.4 (518.4, 640.7)	287.0 (234.0, 346.8)	-49.8% (-59.8, -37.8)
Israel	9.1 (8.2, 10.6)	2.0 (1.6, 2.5)	-77.6% (-83.7, -71.2)	937.0 (848.9, 1073.8)	304.6 (250.7, 360.8)	-67.5% (-73.9, -60.5)
Italy	8.2 (7.5, 8.9)	2.3 (1.9, 2.7)	-71.9% (-78.2, -66.2)	830.7 (766.2, 901.3)	316.3 (273.8, 361.0)	-61.9% (-68.1, -56.3)
Jamaica	28.5 (23.6, 33.9)	21.2 (14.9 , 29.3)	-25.8% (-51.8, 15.1)	2688.7 (2238.0, 3160.0)	2024.0 (1465.9, 2743.7)	-24.7% (-49.6, 13.7)
Japan	2.9 (2.7, 3.2)	0.9 (0.7, 1.0)	-70.8% (-77.5, -65.7)	333.1 (308.6, 360.3)	156.5 (134.4, 180.3)	-53.0% (-59.5, -47.4)
Jordan	28.9 (24.6, 34.0)	12.4 (9.0, 17.4)	-57.2% (-70.1, -38.0)	2745.0 (2356.1, 3192.4)	1279.3 (975.8 1720.8)	-53.4% (-65.8, -34.8)
Kazakhstan	19.6 (16.8, 22.7)	8.0 (5.7, 10.6)	-59.3% (-71.2, -45.6)	1824.5 (1578.5, 2099.3)	795.0 (590.3, 1020.0)	-56.4% (-68.1, -42.9)
Kenya	37.5 (30.9, 44.7)	28.2 (23.0, 34.9)	-24.7% (-43.5, -2.0)	3405.1 (2811.5, 4044.7)	2652.0 (2148.6, 3265.9)	-22.1% (-41.1, 0.8)
Kiribati	41.0 (31.4, 51.6)	23.4 (16.7, 32.7)	-43.1% (-60.3, -16.7)	3711.0 (2860.1, 4648.9)	2154.7 (1567.3, 3001.6)	-41.9% (-59.0, -15.8)
Kuwait	13.3 (11.4, 15.7)	6.8 (5.2, 8.8)	-48.9% (-62.6, -31.7)	1349.0 (1167.6, 1565.7)	789.3 (645.3, 970.7)	-41.5% (-54.2, -25.6)
Kyrgyzstan	35.0 (29.1, 40.9)	14.9 (12.8, 17.1)	-57.4% (-65.9, -46.3)	3195.3 (2663.7, 3723.6)	1416.6 (1224.9, 1610.5)	-55.7% (-64.1, -44.7)
Lao People's Democratic Republic	80.6 (68.5, 94.4)	31.5 (25.0, 38.9)	-60.9% (-70.6, -49.1)	7219.9 (6134.5, 8450.4)	2867.2 (2289.0, 3526.6)	-60.3% (-69.9, -48.5)
Latvia	8.6 (7.7, 9.5)	2.3 (1.5, 3.0)	-73.1% (-82.9, -64.9)	868.2 (789.5, 951.3)	305.1 (234.8, 370.2)	-64.9% (-73.7, -57.0)

Lebanon	28.4 (23.0, 34.1)	6.9 (4.5, 10.3)	-75.5% (-85.1, -62.8)	2659.8 (2182.7, 3182.0)	757.1 (533.4, 1044.2)	-71.5% (-81.0, -59.0)
Lesotho	69.4 (54.9, 87.1)	50.0 (34.0, 67.9)	-27.9% (-56.4, 12.6)	6256.4 (4987.5, 7837.9)	4543.4 (3119.9, 6136.2)	-27.4% (-55.5, 12.5)
Liberia	89.2 (78.1, 100.4)	34.3 (24.5, 47.7)	-61.6% (-72.3, -46.8)	7963.0 (6975.2, 8953.8)	3129.3 (2244.5, 4336.7)	-60.7% (-71.3, -45.8)
Libya	35.7 (28.8, 43.5)	7.4 (5.4, 9.7)	-79.2% (-86.3, -70.9)	3326.1 (2714.0, 4003.1)	796.5 (611.1, 998.3)	-76.1% (-83.1, -67.6)
Lithuania	7.7 (6.8, 8.7)	1.8 (1.2, 2.2)	-77.1% (-84.7, -70.8)	780.1 (690.1, 864.9)	244.8 (192.9, 287.8)	-68.6% (-75.7, -62.1)
Luxembourg	5.6 (4.8, 6.5)	1.6 (1.0, 2.5)	-72.1% (-83.6, -55.2)	595.4 (519.1, 683.9)	235.5 (174.5, 318.1)	-60.4% (-71.1, -45.9)
Madagascar	55.1 (47.1, 63.3)	33.7 (26.4, 42.3)	-38.9% (-52.5, -22.0)	4954.4 (4249.0, 5682.9)	3104.9 (2463.1, 3871.3)	-37.3% (-50.6, -20.2)
Malawi	65.5 (57.5, 74.0)	37.0 (28.4, 48.6)	-43.5% (-56.7, -25.5)	5864.2 (5159.0, 6619.5)	3378.5 (2607.8, 4390.0)	-42.4% (-55.6, -24.2)
Malaysia	12.5 (10.5, 14.9)	4.4 (3.5, 5.5)	-65.2% (-75.7, -51.2)	1234.8 (1056.9, 1437.0)	510.4 (421.3, 616.1)	-58.7% (-69.4, -45.2)
Maldives	46.4 (39.2, 54.5)	14.8 (10.9, 20.5)	-68.1% (-77.7, -54.5)	4263.2 (3628.3, 5011.2)	1425.9 (1075.9, 1937.0)	-66.6% (-75.9, -53.4)
Mali	105.4 (88.5, 123.2)	64.6 (51.5, 81.2)	-38.8% (-52.6, -21.0)	9406.9 (7898.5, 10977.7)	5790.3 (4633.4, 7254.0)	-38.4% (-52.2, -20.8)
Malta	9.6 (8.5, 11.2)	4.9 (3.2, 7.2)	-49.1% (-66.6, -23.2)	985.8 (878.5, 1132.4)	565.6 (417.7, 769.6)	-42.6% (-58.0, -20.2)
Marshall Islands	22.6 (19.2, 26.2)	13.8 (10.5, 18.1)	-38.9% (-53.3, -19.7)	2096.6 (1800.7, 2422.9)	1318.5 (1017.8, 1697.6)	-37.1% (-51.0, -19.1)
Mauritania	68.5 (56.8, 80.4)	36.5 (29.0, 45.8)	-46.7% (-58.9, -31.3)	6178.7 (5157.7, 7233.1)	3377.1 (2718.9, 4195.7)	-45.3% (-57.4, -30.2)
Mauritius	24.4 (22.7, 26.1)	11.8 (8.8, 15.5)	-51.6% (-64.4, -35.7)	2299.1 (2143.1, 2459.8)	1175.6 (900.9, 1504.7)	-48.9% (-60.9, -34.0)
Mexico	29.1 (22.8, 36.3)	10.2 (7.7, 13.2)	-64.9% (-74.8, -51.1)	2654.4 (2085.9, 3285.5)	999.0 (773.0, 1256.3)	-62.4% (-72.2, -48.4)
Micronesia (Federated States of)	23.6 (18.5, 29.1)	8.3 (6.1, 10.9)	-65.0% (-74.5, -52.7)	2195.3 (1744.5, 2689.2)	832.4 (631.8, 1068.4)	-62.1% (-71.6, -49.7)
Monaco	3.8 (2.7, 5.2)	1.3 (0.9, 1.7)	-66.7% (-74.6, -54.9)	437.9 (333.4, 569.3)	220.0 (180.3, 268.4)	-49.7% (-60.5, -34.9)
Mongolia	33.4 (29.0, 38.3)	11.8 (9.3, 15.1)	-64.6% (-72.9, -53.6)	3021.3 (2644.3, 3451.8)	1133.4 (903.5, 1422.7)	-62.5% (-70.9, -51.3)
Montenegro	15.6 (13.5, 17.7)	3.3 (2.6, 4.0)	-79.1% (-83.9, -73.3)	1492.7 (1303.6, 1691.3)	389.3 (320.5, 461.4)	-73.9% (-78.9, -68.1)
Morocco	55.4 (46.8, 64.5)	17.2 (12.5, 22.6)	-69.0% (-77.9, -57.8)	5064.2 (4288.4, 5860.4)	1677.4 (1262.7, 2155.5)	-66.9% (-75.6, -55.7)
Mozambique	72.0 (62.5, 82.4)	38.7 (30.9, 48.1)	-46.2% (-56.6, -32.8)	6437.5 (5590.5, 7360.0)	3500.3 (2810.8, 4334.2)	-45.6% (-56.0, -32.3)
Myanmar	67.8 (55.2, 81.7)	29.5 (21.7, 38.3)	-56.4% (-68.3, -42.3)	6099.6 (4977.7, 7338.8)	2705.4 (1998.9, 3493.2)	-55.6% (-67.5, -41.8)
Namibia	45.4 (38.8, 52.4)	28.0 (21.1, 37.6)	-38.3%(-54.6% to -17.4)	4189.9 (3595.3, 4805.5)	2684.5 (2073.3, 3531.4)	-35.9% (-51.8, -15.6)
Nauru	18.6 (14.4, 23.5)	15.6 (12.1, 20.1)	-16.2% (-35.9, 9.9)	1741.4 (1369.4, 2169.7)	1475. 6 (1161.9, 1886.2)	-15.3% (-34.2, 9.4)
Nepal	80.8 (62.6, 99.4)	29.5 (24.0, 35.8)	-63.5% (-72.9, -49.7)	7375.7 (5732.4, 9038.9)	2840.0 (2349.3, 3381.5)	-61.5% (-70.6, -47.7)

Netherlands	5.6 (5.0, 6.3)	2.9 (2.3, 3.7)	-47.8% (-60.5, -32.0)	597.7 (534.7, 659.6)	365.3 (300.2, 439.5)	-38.9% (-50.2, -25.1)
New Zealand	6.5 (6.1, 7.0)	3.2 (2.5, 4.0)	-51.0% (-62.4, -37.3)	674.6 (626.6, 727.5)	402.3 (332.9, 481.7)	-40.4% (-51.0, -28.0)
Nicaragua	34.0 (30.1, 38.0)	8.8 (6.7, 11.3)	-74.0% (-80.9, -65.6)	3114.6 (2773.6, 3468.3)	881.2 (683.0 1098.0)	-71.7% (-78.7, -63.4)
Niger	70.7 (59.2, 83.2)	44.7 (37.9, 53.0)	-36.8% (-50.3, -20.1)	6319.3 (5307.8, 7436.8)	4029.5 (3432.1, 4751.6)	-36.2% (-49.7, -19.6)
Nigeria	72.8 (63.5, 81.9)	56.6 (45.5, 70.9)	-22.3% (-38.7, 0.3)	6498.3 (5661.6, 7299.8)	5092.5 (4100.7, 6363.1)	-21.6% (-38.1, 0.9)
Niue	15.5 (11.8, 19.7)	11.6 (8.7, 15.4)	-24.9% (-43.4, -1.9)	1481.0 (1149.0, 1856.9)	1145.0 (878.8, 1474.1)	-22.7% (-40.4, -1.1)
North Macedonia	29.8 (27.2, 32.4)	9.6 (7.8, 11.7)	-67.6% (-74.0, -59.3)	2757.2 (2519.7, 2989.5)	964.9 (803.1, 1152.6)	-65.0% (-71.2, -57.3)
Northern Mariana Islands	7.7 (5.6, 10.1)	6.3 (4.8, 8.1)	-17.5% (-35.8, 3.6)	797.8 (613.5, 1016.8)	683.7 (538.8, 845.5)	-14.3% (-31.0, 5.0)
Norway	5.2 (4.5, 5.7)	1.5 (1.1, 1.8)	-71.6% (-79.4, -64.5)	561.1 (497.2, 615.8)	226.3 (184.9, 265.9)	-59.7% (-65.8, -53.4)
Oman	27.4 (20.8, 35.3)	7.8 (6.5, 9.3)	-71.4% (-79.4, -61.0)	2579.1 (1998.0, 3276.5)	844.3 (719.7, 978.7)	-67.3% (-75.7, -56.1)
Pakistan	92.0 (81.5, 102.4)	69.4 (56.6, 83.8)	-24.5% (-40.3, -4.8)	8239.2 (7312.7, 9162.3)	6301.6 (5168.6, 7591.5)	-23.5% (-39.2, -3.8)
Palau	19.9 (15.1, 25.0)	8.5 (5.9, 11.7)	-57.1% (-69.3, -40.6)	1862.9 (1439.0, 2334.7)	860.5 (633.7, 1147.4)	-53.8% (-65.6, -38.0)
Palestine	34.5 (28.8, 40.9)	10.8 (8.0, 14.4)	-68.7% (-77.3, -57.3)	3183.7 (2683.3, 3760.1)	1091.2 (851.6, 1425.3)	-65.7% (-74.3, -54.7)
Panama	16.2 (13.7, 19.0)	8.3 (5.8, 11.2)	-48.6% (-64.2, -28.8)	1540.9 (1320.2, 1791.0)	839.8 (623.1, 1098.1)	-45.5% (-60.2, -26.7)
Papua New Guinea	33.3 (26.6, 40.4)	26.6 (19.5, 35.8)	-20.0% (-40.5, 7.3)	3020.1 (2426.9, 3650.5)	2431.0 (1801.1, 3251.2)	-19.5% (-39.5, 7.2)
Paraguay	24.8 (20.9, 29.1)	7.2 (4.8, 10.2)	-70.9% (-81.4, -58.2)	2351.1 (1997.0, 2733.0)	776.6 (558.5, 1041.8)	-67.0% (-77.0, -55.1)
Peru	36.5 (31.1, 42.8)	10.6 (7.3, 14.6)	-71.1% (-81.0, -58.2)	3333.3 (2834.5, 3899.2)	1036.3 (743.0, 1397.7)	-68.9% (-78.7, -56.2)
Philippines	31.2 (26.4, 36.5)	16.9 (12.3, 22.3)	-46.0% (-62.7, -24.6)	2835.2 (2407.1, 3310.6)	1583.9 (1181.1, 2060.0)	-44.1% (-60.4, -23.2)
Poland	15.2 (14.1, 16.8)	2.9 (2.1, 3.9)	-81.1% (-87.0, -74.1)	1444.1 (1345.1, 1592.1)	364.8 (283.5, 459.2)	-74.7% (-80.8, -68.1)
Portugal	10.1 (8.8, 11.8)	2.1 (1.6, 2.7)	-79.2% (-85.5, -72.0)	994.0 (878.8, 1145.6)	287.6 (231.1, 348.3)	-71.1% (-77.8, -63.9)
Puerto Rico	17.7 (16.5, 19.2)	7.9 (5.8, 10.5)	-55.4% (-67.4, -39.8)	1724.7 (1607.3, 1870.6)	855.5 (671.0, 1090.0)	-50.4% (-61.4, -36.0)
Qatar	22.1 (17.4, 27.9)	5.8 (4.4, 7.7)	-73.7% (-81.4, -63.5)	2154.6 (1734.5, 2668.0)	712.9 (581.3, 882.8)	-66.9% (-75.1, -56.7)
Republic of Korea	7.1 (5.8, 8.9)	2.2 (1.8, 2.6)	-69.2% (-77.3, -60.8)	712.6 (601.8, 878.8)	278.1 (240.1, 322.1)	-61.0% (-69.8, -52.2)
Republic of Moldova	15.7 (12.8, 19.2)	8.7 (6.7, 11.3)	-44.8% (-59.5, -25.3)	1498.7 (1239.8, 1818.2)	864.9 (681.8, 1100.9)	-42.3% (-56.3, -23.9)
Romania	11.5 (10.1, 13.3)	5.2 (4.1, 6.4)	-54.9% (-65.2, -42.3)	1115.4 (980.8, 1284.2)	562.3 (462.0, 673.9)	-49.6% (-59.5, -37.8)
Russian Federation	14.3 (13.4, 16.0)	3.5 (2.7, 4.3)	-75.8% (-81.3, -69.8)	1356.0 (1272.7, 15033)	405.2 (336.3, 481.4)	-70.1% (-75.6, -64.4)

Rwanda	59.7 (51.2, 69.5)	27.9 (22.5, 34.8)	-53.2% (-63.6, -39.3)	5340.3 (4589.1, 6211.1)	2554.5 (2075.8, 3170.0)	-52.2% (-62.5, -38.3)
Saint Kitts and Nevis	33.1 (29.2, 37.3)	15.8 (12.3, 20.0)	-52.4% (-64.0, -38.0)	3079.7 (2738.4, 3460.7)	1531.2 (1219.1, 1919.2)	-50.3% (-61.3, -36.1)
Saint Lucia	23.9 (19.4, 28.6)	16.8 (11.5, 23.9)	-29.6% (-53.3, 5.6)	2258.5 (1874.2, 2685.0)	1629.3 (1153.8, 2254.6)	-27.9% (-50.5, 4.7)
Saint Vincent and the Grenadines	30.3 (23.4, 37.9)	15.3 (10.7, 21.2)	-49.6% (-66.8, -25.4)	2821.6 (2222.5, 3492.9)	1482.0 (1073.9, 2013.6)	-47.5% (-64.4, -23.6)
Samoa	22.3 (16.1, 29.6)	7.7 (5.1, 10.9)	-65.4% (-77.8, -46.0)	2086.5 (1545.6, 2736.3)	799.7 (567.8, 1082.8)	-61.7% (-74.2, -42.9)
San Marino	5.8 (4.2, 7.6)	2.5 (1.9, 3.2)	-56.0% (-70.1, -34.5)	615.3 (470.9, 782.3)	326.8 (265.2, 399.1)	-46.9% (-60.6, -27.7)
Sao Tome and Principe	32.8 (28.5, 37.4)	17.9 (12.8, 24.4)	-45.3% (-62.6, -23.5)	3017.0(2633.6, 3433.9)	1772.1 (1300.8, 2344.4)	-41.3% (-58.2, -19.8)
Saudi Arabia	32.6 (24.2, 41.5)	3.9 (2.8, 5.1)	-88.1% (-91.8, -83.4)	3025.2 (2261.6, 3822.6)	493.5 (393.7, 608.3)	-83.7% (-88.0, -78.1)
Senegal	60.9 (52.6, 70.2)	41.3 (33.9, 50.4)	-32.1% (-45.8, -14.2)	5480.7 (4744.8, 6303.6)	3800.8 (3143.2, 4606.8)	-30.7% (-44.3, -12.8)
Serbia	24.6 (22.4, 27.4)	3.7 (2.9, 4.6)	-84.9% (-88.8, -80.6)	2307.9 (2104.2, 2556.5)	438.6 (358.2, 533.3)	-81.0% (-84.9, -76.6)
Seychelles	19.1 (16.3, 22.3)	9.4 (6.9, 12.3)	-50.9% (-64.3, -32.7)	1828.1 (1571.8, 2114.7)	949.6 (724.9, 1221.0)	-48.1% (-60.8, -31.3)
Sierra Leone	96.8 (75.3, 119.4)	45.2 (36.0, 56.6)	-53.3% (-60.9, -44.5)	8626.3 (6721.8, 10637.0)	4052.5 (3236.3, 5060.9)	-53.0% (-60.5, -44.3)
Singapore	4.8 (4.3, 5.7)	1.0 (0.7, 1.4)	-79.8% (-87.7, -69.9)	523.2 (466.6, 610.2)	186.1 (147.2, 231.4)	-64.4% (-73.4, -54.5)
Slovakia	12.4 (11.4, 13.7)	3.8 (2.8, 5.1)	-69.1% (-77.6, -58.1)	1205.4 (1102.9, 1324.7)	435.8 (340.5, 549.2)	-63.8% (-72.2, -53.7)
Slovenia	7.6 (6.8, 8.4)	1.5 (1.0, 2.1)	-80.4% (-87.2, -73.0)	779.7 (702.0, 851.9)	245.0 (190.0 302.8)	-68.6% (-75.5, -61.0)
Solomon Islands	23.3 (17.0, 30.1)	11.3 (7.4, 15.9)	-51.4% (-64.3, -34.6)	2143.8 (1585.1, 2741.8)	1084.5 (741.2, 1504.9)	-49.4% (-61.6, -32.8)
Somalia	62.5 (51.2, 73.4)	42.1 (31.6, 55.4)	-32.6% (-49.4, -9.6)	5589.5 (4584.1, 6557.1)	3798.6 (2857.5, 4975.9)	-32.0% (-48.8, -9.4)
South Africa	41.9 (33.5, 51.9)	33.7 (25.8, 43.8)	-19.5% (-40.9, 11.7)	3835.9 (3085.8, 4716.1)	3198.5 (2490.5, 4122.5)	-16.6% (-37.7, 13.8)
South Sudan	68.2 (56.9, 81.7)	49.1 (39.1, 61.4)	-28.0% (-44.7, -8.0)	6100.7 (5102.6, 7300.4)	4428.2 (3541.4, 5502.4)	-27.4% (-44.0, -7.7)
Spain	6.6 (5.9, 7.5)	2.2 (1.7, 2.6)	-67.3% (-75.5, -58.5)	692.2 (626.6, 778.0)	286.2 (240.4, 336.7)	-58.6% (-66.6, -50.4)
Sri Lanka	20.6 (17.6, 23.8)	6.3 (4.2, 9.0)	-69.4% (-80.4, -53.9)	1951.3 (1682.7, 2249.7)	672.0 (489.6, 919.1)	-65.6% (-76.1, -51.0)
Sudan	82.4 (67.7, 98.8)	34.6 (26.6, 45.0)	-58.1% (-68.4, -44.8)	7387.2 (6081.0, 8846.1)	3152.5 (2452.5, 4089.8)	-57.3% (-67.7, -44.2)
Suriname	48.7 (42.5, 55.4)	27.2 (19.2, 37.7)	-44.3% (-61.6, -21.7)	4474.5 (3911.9, 5086.3)	2555.6 (1842.3, 3498.3)	-42.9% (-59.2, -20.7)
Sweden	3.8 (3.4, 4.2)	1.6 (1.2, 2.0)	-57.0% (-69.3, -46.1)	414.9 (375.2, 454.9)	226.1 (182.6, 263.7)	-45.5% (-55.6, -36.0)
Switzerland	4.8 (4.3, 5.6)	3.0 (2.6, 35)	-38.0% (-49.8, -23.9)	538.7 (478.7, 611.1)	380.9 (334.2, 436.6)	-29.3% (-40.6, -16.2)
Syrian Arab Republic	38.0 (29.9, 46.9)	8.0 (6.0, 10.1)	-79.0% (-84.6, -71.5)	3511.3 (2785.0, 4317.0)	832.3 (656.1, 1026.6)	-76.3% (-82.1, -68.7)

Taiwan (Province of China)	2.8 (2.4, 3.1)	3.2 (2.6, 4.0)	15.4% (-9.6, 51.3)	345.4 (301.4, 387.7)	393.1 (332.9, 471.3)	13.8% (-5.5, 41.1)
Tajikistan	33.5 (28.0 39.1)	18.1 (14.5, 22.4)	-46.0% (-58.8, -29.2)	3051.5 (2558.8, 3545.9)	1699.8 (1385.9, 2085.1)	-44.3% (-56.9, -27.6)
Thailand	22.1 (17.9, 26.9)	5.1 (3.9, 6.5)	-77.0% (-83.7, -68.3)	2071.5 (1703.9, 2504.0)	558.2 (447.8, 693.4)	-73.1% (-80.0, -64.3)
Timor-Leste	54.8 (46.6, 64.0)	26.0 (20.7, 32.4)	-52.6% (-64.0, -38.6)	4981.3 (4266.5, 5792.8)	2424.1 (1944.7, 3016.3)	-51.3% (-62.6, -37.5)
Togo	68.3 (59.9, 77.2)	40.2 (32.3, 50.2)	-41.1% (-54.5, -24.6)	6132.7 (5377.0, 6913.2)	3659.8 (2953.7, 4551.3)	-40.3% (-53.5, -23.9)
Tokelau	16.5 (12.8, 21.2)	3.9 (2.8, 5.2)	-76.2% (-82.8, -67.7)	1578.0 (1245.6, 1997.0)	464.5 (358.9, 582.4)	-70.6% (-77.6, -61.8)
Tonga	17.3 (14.0, 21.1)	8.8 (6..1, 12.2)	-49.0% (-65.3, -27.1)	1659.8 (1349.4, 1996.2)	904.9 (666.6, 1207.0)	-45.5% (-61.1, -24.9)
Trinidad and Tobago	29.7 (24.5, 35.4)	15.6 (10.9, 21.7)	-47.5% (-64.4, -22.5)	2773.8 (2306.7, 3282.5)	1519.0 (1099.8, 2070.6)	-45.2% (-61.8, -21.4)
Tunisia	42.0 (33.3, 52.7)	9.6 (7.4, 12.1)	-77.1% (-83.8, -67.8)	3859.8 (3077.4, 4810.5)	983.5 (782.0, 1205.8)	-74.5% (-81.5, -65.1)
Turkey	55.8 (45.2, 67.2)	12.7(9.8, 16.0)	-77.3% (-83.8, -69.7)	5106.9 (4163.4, 6088.0)	1285.9 (1032.1, 1585.8)	-74.8% (-81.3, -66.7)
Turkmenistan	26.5(22.0, 31.5)	16.4 (13.5, 20.0)	-37.9% (-49.9, -23.6)	2405.5 (2010.6, 2850.7)	1530.3 (1270.7, 1850.2)	-36.4% (-48.1, -22.2)
Tuvalu	40.6 (32.9, 49.8)	9.0 (6.4, 12.6)	-77.7% (-84.2, -68.9)	3686.8 (2994.9, 4503.3)	902.3 (667.7, 1213.1)	-75.5% (-81.9, -66.5)
Uganda	52.6 (46.9, 58.5)	36.1 (28.2, 45.2)	-31.4% (-46.6, -13.0)	4720.3 (4205.4, 5244.6)	3287.7 (2587.3, 4100.5)	-30.4% (-45.4, -11.9)
Ukraine	12.6 (10.3, 15.3)	5.2 (4.1, 6.5)	-58.7% (-70.8, -42.2)	1196.3 (994.2, 1439.7)	544.4 (440.3, 664.1)	-54.5% (-66.6, -38.7)
United Arab Emirates	13.3 (9.5, 17.7)	2.5 (1.6, 3.8)	-81.0% (-89.3, -69.2)	1332.4 (994.9, 1732.0)	391.3 (300.4, 505.3)	-70.6% (-80.2, -57.6)
United Kingdom	7.1 (6.4, 7.6)	3.6 (3.0, 4.4)	-48.9% (-57.8, -38.4)	738.4 (669.3, 789.4)	437.1 (370.5, 509.5)	-40.8% (-48.5, -32.0)
United Republic of Tanzania	52.7 (44.1, 61.0)	35.8 (27.7, 46.1)	-32.1% (-49.0, -12.0)	4734.9 (3974.9, 5474.1)	3261.4 (2537.7, 4188.1)	-31.1% (-47.9, -11.0)
United States of America	9.0 (8.5, 9.5)	5.5 (5.0, 6.0)	-38.8% (-44.9, -32.2)	949.7 (894.2, 1010.2)	647.7 (589.6, 708.9)	-31.8% (-37.4, -26.0)
United States Virgin Islands	17.8 (15.1, 20.6)	6.6 (4.6, 9.1)	-63.1% (-75.1, -48.3)	1731.1 (1490.5, 1978.6)	728.8 (540.8, 959.4)	-57.9% (-69.3, -43.4)
Uruguay	19.0 (17.0, 20.7)	5.9 (4.2, 8.1)	-69.0% (-78.4, -57.2)	1817.9 (1625.7, 1975.6)	650.9 (495.5, 846.7)	-64.2% (-73.2, -52.7)
Uzbekistan	23.0 (20.1, 26.3)	14.5 (11.5, 18.5)	-36.7% (-51.5, -16.7)	2109.6 (1860.0, 2403.7)	1371.5 (1103.6, 1720.6)	-35.0% (-49.2, -15.6)
Vanuatu	23.8 (18.4, 29.9)	16.0 (12.0, 20.7)	-32.6% (-50.1, -10.0)	2204.8 (1719.8, 2746.4)	1519.0 (1172.0, 1933.1)	-31.1% (-48.2, -9.2)
Venezuela (Bolivarian Republic of)	23.8 (21.2, 25.7)	13.1 (9.2, 18.3)	-44.9% (-61.8, -23.4)	2228.1 (1997.4, 2405.0)	1275.8 (935.6, 1734.1)	-42.7% (-58.8, -22.3)
Viet Nam	31.2 (27.0, 36.0)	8.9 (6.5, 11.9)	-71.6% (-80.3, -59.5)	2887.0 (2514.5, 3314.7)	874.6 (656.3, 1146.6)	-69.7% (-78.2, -58.0)
Yemen	77.5 (61.5, 95.8)	39.1 (30.5, 48.7)	-49.5% (-61.4, -34.9)	7016.4 (5596.0, 8674.4)	3635.0 (2869.4, 4485.7)	-48.2% (-60.1, -33.7)
Zambia	48.3 (42.3, 54.3)	31.2 (23.5, 42.3)	-35.4% (-52.8, -12.0)	4340.1 (3809.3, 4874.3)	2870.1 (2181.6, 3845.0)	-33.9% (-51.2, -10.5)

Zimbabwe	40.1 (36.0, 44.1)	40.7 (31.8, 52.2)	1.6% (-22.2, 31.1)	3664.6 (3304.3, 4027.2)	3745.6 (2947.5, 4769.3)	2.2% (-21.0,o 31.3)

DALY= disability-adjusted life year; PBLBW= preterm birth and low birth weight; ASR= age standardized rate; PAF= population attributable fraction; SDI= Socio-demographic Index.

*Per 100 000 people.

Table S4. Deaths and DALYs attributable to PBLBW in 2019 and percentage change from 1990 to 2019.

Cause of death or DALYs	Deaths				DALYs			
	2019 ASR*, n (95% UI)	Percentage change in ASR (1990–2019), % (95% CI)	2019 age standardized PAF, % (95% CI)	Percentage change in age standardized PAF (1990–2019), % (95% CI)	2019 ASR*, n (95% UI)	Percentage change in ASR (1990–2019), % (95% CI)	2019 age standardized PAF, % (95% CI)	Percentage change in age standardized PAF (1990–2019), % (95% CI)
Diarrheal diseases	0.52 (0.42, 0.64)	-77.8% (-84.1, -67.7)	2.5% (1.7, 3.4)	-37.6% (-54.7, -15.1)	46.19 (37.38, 56.89)	-77.8% (-84.1, -67.7)	4.1% (3.3, 4.8)	-37.2% (-49.9, -16.2)
Encephalitis	0.02 (0.01, 0.02)	-34.1% (-55.6, 2.0)	1.5% (1.1, 1.8)	21.8% (-12.9, 58.0)	1.53 (1.18, 1.96)	-34.1% (-55.6, 2.0)	2.3% (1.8, 2.8)	43.9% (3.0, 92.7)
Lower respiratory infections	2.41 (1.99, 2.92)	-61.4% (-69.6, -51.2)	7.0% (6.0, 8.1)	-25.0% (-36.7, -11.6)	214.49 (176.59, 259.64)	-61.4% (-69.6, -51.2)	15.5% (14.1, 16.9)	2.9% (-9.9, 16.6)
Meningitis	0.17 (0.13, 0.22)	-51.4% (-61.0, -38.4)	5.1% (4.5, 5.9)	10.3% (-4.5, 27.8)	14.9 (11.92, 19.23)	-51.4% (-61.0, -38.4)	6.4% (5.7, 7.3)	13.4% (-1.6, 30.6)
Neonatal disorders	24.72 (21.05, 29.33)	-37.0% (-46.3, -25.0)	85.1% (83.7, 86.3)	-0.6% (-1.6, 0.4)	2324.31 (1990.74, 2734.08)	-34.9% (-44.1, -23.2)	82.2% (80.6, 83.7)	-3.4% (-4.9, -2.1%)
Otitis media	0 (0, 0.01)	-69.1% (-90.9, -40.2)	22.7% (2.1, 48.5)	73.9% (-24.3, 206.8)	0.22 (0.01, 0.86)	-69.1% (-90.9, -40.2)	0.7% (0.0, 2.8)	-61.6% (-88.8, -26.9)
Sudden infant death syndrome	0.03 (0.02, 0.06)	-50.1% (-66.4, -15.2)	7.6% (5.8, 10.1)	9.4% (-19.3, 37.9)	2.66 (1.48, 5.13)	-50.1% (-66.4, -15.2)	7.6% (5.8, 10.2)	9.4% (-19.3, 37.8)
Upper respiratory infections	0 (0, 0)	-83.5% (-88.1, -64.5)	0.9% (0.5, 1.3)	-13.3% (-51.7, 19.6)	0.11 (0.05, 0.17)	-83.5% (-88.1, -64.5)	0.1% (0.1, 0.2)	-76.3% (-82.9, -62.4)

DALY= disability-adjusted life year; PBLBW= preterm birth and low birth weight; ASR= age standardized rate; PAF= population attributable fraction.

*Per 100 000 people.

Table S5. The proportions of PBLBW-related age-standardized DALYs in 2019 among the global and 21 GBD regions.

Location	Sex	Cause	Val (%)	Upper (%)	Lower (%)
Global	Male	Upper respiratory infections	0.2	0.3	0.1
Global	Female	Upper respiratory infections	0.1	0.2	0
Global	Male	Otitis media	0.9	4.1	0
Global	Female	Otitis media	0.5	2.6	0
Global	Male	Neonatal disorders	83	84.6	81
Global	Female	Neonatal disorders	81.1	83	79
Global	Male	Diarrheal diseases	4.2	5	3.3
Global	Female	Diarrheal diseases	4	5	2.8
Global	Male	Meningitis	6.7	7.9	5.8
Global	Female	Meningitis	5.9	6.9	5.1
Global	Male	Encephalitis	2.2	2.7	1.7
Global	Female	Encephalitis	2.5	3.1	1.8
Global	Male	Lower respiratory infections	15.2	16.9	13.7
Global	Female	Lower respiratory infections	15.5	17.1	14
Global	Male	Sudden infant death syndrome	7.5	10.2	5.7
Global	Female	Sudden infant death syndrome	7.7	10.2	5.9
Andean Latin America	Male	Upper respiratory infections	0.1	0.2	0
Andean Latin America	Female	Upper respiratory infections	0.1	0.2	0
Andean Latin America	Male	Otitis media	0.1	0.6	0
Andean Latin America	Female	Otitis media	0	0	0
Andean Latin America	Male	Neonatal disorders	68.7	73.1	64.2
Andean Latin America	Female	Neonatal disorders	66.1	70.2	61.4
Andean Latin America	Male	Lower respiratory infections	4.2	5.4	3.2
Andean Latin America	Female	Lower respiratory infections	4.3	5.4	3.3

Andean Latin America	Male	Diarrheal diseases	0.9	1.5	0.5
Andean Latin America	Female	Diarrheal diseases	1	1.7	0.5
Andean Latin America	Male	Meningitis	4.4	6	3.1
Andean Latin America	Female	Meningitis	5.1	6.9	3.7
Andean Latin America	Male	Encephalitis	2.6	4.1	1.5
Andean Latin America	Female	Encephalitis	3.5	5.6	2.2
Andean Latin America	Male	Sudden infant death syndrome	5.6	9.1	3.5
Andean Latin America	Female	Sudden infant death syndrome	7.3	10.8	5.1
Australasia	Male	Upper respiratory infections	0	0	0
Australasia	Female	Upper respiratory infections	0	0	0
Australasia	Male	Otitis media	0	0.1	0
Australasia	Female	Otitis media	0	0	0
Australasia	Male	Neonatal disorders	80.3	82.5	77.9
Australasia	Female	Neonatal disorders	80.5	82.6	78.4
Australasia	Male	Lower respiratory infections	1.8	2.3	1.3
Australasia	Female	Lower respiratory infections	2.3	2.9	1.8
Australasia	Male	Diarrheal diseases	0.4	0.6	0.2
Australasia	Female	Diarrheal diseases	0.4	0.6	0.2
Australasia	Male	Meningitis	6.2	7.9	4.7
Australasia	Female	Meningitis	6.2	7.7	4.8
Australasia	Male	Encephalitis	1.2	1.8	0.8
Australasia	Female	Encephalitis	1.1	1.7	0.8
Australasia	Male	Sudden infant death syndrome	5.2	7.4	3.7
Australasia	Female	Sudden infant death syndrome	6.1	8.9	4.2
Caribbean	Male	Otitis media	1.3	7.2	0
Caribbean	Female	Otitis media	1.3	7.6	0

Caribbean	Male	Meningitis	13.6	21.8	7.9
Caribbean	Female	Meningitis	16.1	24.8	9.6
Caribbean	Male	Neonatal disorders	82.4	84.9	79.4
Caribbean	Female	Neonatal disorders	78.7	81.5	75.2
Caribbean	Male	Upper respiratory infections	0	0.1	0
Caribbean	Female	Upper respiratory infections	0	0.1	0
Caribbean	Male	Lower respiratory infections	13.7	19.4	9.3
Caribbean	Female	Lower respiratory infections	15.1	20.8	10.3
Caribbean	Male	Diarrheal diseases	6.9	11.9	3.4
Caribbean	Female	Diarrheal diseases	8.1	15	3.4
Caribbean	Male	Encephalitis	3.7	6.1	2.1
Caribbean	Female	Encephalitis	3	4.8	1.8
Caribbean	Male	Sudden infant death syndrome	8.9	16.5	3.9
Caribbean	Female	Sudden infant death syndrome	14.2	22.5	8.1
Central Asia	Male	Diarrheal diseases	2.5	3.8	1.6
Central Asia	Female	Diarrheal diseases	2.2	3.4	1.4
Central Asia	Male	Encephalitis	2.4	3.2	1.7
Central Asia	Female	Encephalitis	2.6	4.1	1.5
Central Asia	Male	Sudden infant death syndrome	8.9	11.9	6.3
Central Asia	Female	Sudden infant death syndrome	7.2	9.6	5.5
Central Asia	Male	Otitis media	0	0	0
Central Asia	Female	Otitis media	0	0	0
Central Asia	Male	Meningitis	4.1	5.1	3.2
Central Asia	Female	Meningitis	3.9	5.1	3
Central Asia	Male	Neonatal disorders	73.8	76.4	70.8
Central Asia	Female	Neonatal disorders	65.1	68	62

Central Asia	Male	Lower respiratory infections	11.4	12.8	10.1
Central Asia	Female	Lower respiratory infections	9.6	10.7	8.5
Central Asia	Male	Upper respiratory infections	2.3	3.7	0.9
Central Asia	Female	Upper respiratory infections	1.8	3.1	1
Central Europe	Male	Lower respiratory infections	3	3.7	2.5
Central Europe	Female	Lower respiratory infections	4.3	5	3.7
Central Europe	Male	Diarrheal diseases	0.7	1	0.4
Central Europe	Female	Diarrheal diseases	0.4	0.7	0.3
Central Europe	Male	Encephalitis	2.3	2.9	1.7
Central Europe	Female	Encephalitis	2.2	2.8	1.5
Central Europe	Male	Upper respiratory infections	0	0.1	0
Central Europe	Female	Upper respiratory infections	0	0.1	0
Central Europe	Male	Sudden infant death syndrome	6.7	8	5.7
Central Europe	Female	Sudden infant death syndrome	7.9	9.5	6.6
Central Europe	Male	Neonatal disorders	77.1	79.7	74.3
Central Europe	Female	Neonatal disorders	76	78.5	73.3
Central Europe	Male	Otitis media	0.1	0.3	0
Central Europe	Female	Otitis media	0	0.1	0
Central Europe	Male	Meningitis	4.8	5.8	4
Central Europe	Female	Meningitis	6.4	7.6	5.4
Central Latin America	Male	Lower respiratory infections	6.8	7.7	5.9
Central Latin America	Female	Lower respiratory infections	5.9	6.6	5.1
Central Latin America	Male	Diarrheal diseases	1.5	2.1	1.1
Central Latin America	Female	Diarrheal diseases	1.2	1.7	0.9
Central Latin America	Male	Sudden infant death syndrome	12.8	16.1	10
Central Latin America	Female	Sudden infant death syndrome	12.3	15.7	9.4

Central Latin America	Male	Upper respiratory infections	0.3	0.5	0.2
Central Latin America	Female	Upper respiratory infections	0.2	0.4	0.1
Central Latin America	Male	Otitis media	0	0	0
Central Latin America	Female	Otitis media	0	0	0
Central Latin America	Male	Neonatal disorders	75.6	77.9	72.7
Central Latin America	Female	Neonatal disorders	70.8	73.9	67
Central Latin America	Male	Meningitis	4.2	5	3.5
Central Latin America	Female	Meningitis	3.9	4.7	3.3
Central Latin America	Male	Encephalitis	2.4	3.4	1.6
Central Latin America	Female	Encephalitis	2.3	3.1	1.6
Central Sub-Saharan Africa	Male	Sudden infant death syndrome	5.6	10.8	2.8
Central Sub-Saharan Africa	Female	Sudden infant death syndrome	5.5	11.4	2.8
Central Sub-Saharan Africa	Male	Meningitis	5.6	9.6	3.4
Central Sub-Saharan Africa	Female	Meningitis	4.5	7.1	2.8
Central Sub-Saharan Africa	Male	Encephalitis	1.8	3.3	1.1
Central Sub-Saharan Africa	Female	Encephalitis	1.7	3.2	1
Central Sub-Saharan Africa	Male	Upper respiratory infections	0.2	0.4	0
Central Sub-Saharan Africa	Female	Upper respiratory infections	0.1	0.3	0
Central Sub-Saharan Africa	Male	Otitis media	2.9	13.1	0.1
Central Sub-Saharan Africa	Female	Otitis media	1.8	8.4	0
Central Sub-Saharan Africa	Male	Neonatal disorders	81.9	85.2	78.2
Central Sub-Saharan Africa	Female	Neonatal disorders	80	83.7	75.8
Central Sub-Saharan Africa	Male	Lower respiratory infections	7.4	10.7	4.8
Central Sub-Saharan Africa	Female	Lower respiratory infections	7.9	11.7	5
Central Sub-Saharan Africa	Male	Diarrheal diseases	3.1	5.4	1.4
Central Sub-Saharan Africa	Female	Diarrheal diseases	2.9	6.4	1.3

East Asia	Male	Upper respiratory infections	0.1	0.3	0.1
East Asia	Female	Upper respiratory infections	0.1	0.3	0
East Asia	Male	Otitis media	0	0	0
East Asia	Female	Otitis media	0	0	0
East Asia	Male	Neonatal disorders	66.3	70	62
East Asia	Female	Neonatal disorders	64.7	69	59.9
East Asia	Male	Lower respiratory infections	8.1	9.3	6.8
East Asia	Female	Lower respiratory infections	10.4	11.7	9.1
East Asia	Male	Meningitis	2.8	3.2	2.3
East Asia	Female	Meningitis	3.6	4.2	2.8
East Asia	Male	Encephalitis	1.5	1.8	1.2
East Asia	Female	Encephalitis	1.7	2	1.4
East Asia	Male	Sudden infant death syndrome	7.3	8.7	5.8
East Asia	Female	Sudden infant death syndrome	7.4	8.9	6.1
East Asia	Male	Diarrheal diseases	0.8	1.1	0.5
East Asia	Female	Diarrheal diseases	0.8	1.1	0.5
Eastern Europe	Male	Lower respiratory infections	2.1	2.7	1.7
Eastern Europe	Female	Lower respiratory infections	4.5	5.4	3.7
Eastern Europe	Male	Diarrheal diseases	0.5	0.7	0.3
Eastern Europe	Female	Diarrheal diseases	0.4	0.6	0.3
Eastern Europe	Male	Sudden infant death syndrome	4.7	5.9	3.7
Eastern Europe	Female	Sudden infant death syndrome	5.9	7.7	4.3
Eastern Europe	Male	Meningitis	2.6	3.1	2.1
Eastern Europe	Female	Meningitis	3.4	4.1	2.9
Eastern Europe	Male	Encephalitis	1.8	2.2	1.2
Eastern Europe	Female	Encephalitis	2	2.6	1.2

Eastern Europe	Male	Upper respiratory infections	0.3	0.5	0.1
Eastern Europe	Female	Upper respiratory infections	0.2	0.4	0.1
Eastern Europe	Male	Otitis media	0	0	0
Eastern Europe	Female	Otitis media	0	0	0
Eastern Europe	Male	Neonatal disorders	65.7	68.6	62.5
Eastern Europe	Female	Neonatal disorders	62.7	65.9	59
Eastern Sub-Saharan Africa	Male	Lower respiratory infections	7.8	9.5	6.4
Eastern Sub-Saharan Africa	Female	Lower respiratory infections	8.6	10.2	7.1
Eastern Sub-Saharan Africa	Male	Sudden infant death syndrome	4.7	7.4	3
Eastern Sub-Saharan Africa	Female	Sudden infant death syndrome	5.1	8.3	3.4
Eastern Sub-Saharan Africa	Male	Upper respiratory infections	0.2	0.5	0
Eastern Sub-Saharan Africa	Female	Upper respiratory infections	0.2	0.5	0
Eastern Sub-Saharan Africa	Male	Otitis media	4.3	19.1	0.1
Eastern Sub-Saharan Africa	Female	Otitis media	2.4	12.1	0
Eastern Sub-Saharan Africa	Male	Neonatal disorders	79.5	81.7	76.9
Eastern Sub-Saharan Africa	Female	Neonatal disorders	76.6	79.2	73.8
Eastern Sub-Saharan Africa	Male	Diarrheal diseases	3	4.4	2.2
Eastern Sub-Saharan Africa	Female	Diarrheal diseases	3.2	4.7	1.9
Eastern Sub-Saharan Africa	Male	Meningitis	4.9	6.4	3.9
Eastern Sub-Saharan Africa	Female	Meningitis	4.8	6	3.7
Eastern Sub-Saharan Africa	Male	Encephalitis	4.1	5.7	3
Eastern Sub-Saharan Africa	Female	Encephalitis	4.8	6.5	3.5
High-income Asia Pacific	Male	Diarrheal diseases	0.9	1.2	0.7
High-income Asia Pacific	Female	Diarrheal diseases	1	1.4	0.7
High-income Asia Pacific	Male	Meningitis	2.6	3.3	2
High-income Asia Pacific	Female	Meningitis	3.1	3.9	2.4

High-income Asia Pacific	Male	Encephalitis	1.2	1.9	0.7
High-income Asia Pacific	Female	Encephalitis	1.2	1.8	0.9
High-income Asia Pacific	Male	Sudden infant death syndrome	4.9	6.8	3.7
High-income Asia Pacific	Female	Sudden infant death syndrome	5.5	7.2	4.2
High-income Asia Pacific	Male	Upper respiratory infections	0	0	0
High-income Asia Pacific	Female	Upper respiratory infections	0	0	0
High-income Asia Pacific	Male	Otitis media	0	0	0
High-income Asia Pacific	Female	Otitis media	0	0	0
High-income Asia Pacific	Male	Neonatal disorders	67.2	70.4	63.6
High-income Asia Pacific	Female	Neonatal disorders	68.4	71.4	65.1
High-income Asia Pacific	Male	Lower respiratory infections	0.4	0.5	0.3
High-income Asia Pacific	Female	Lower respiratory infections	0.9	1.1	0.7
High-income North America	Male	Sudden infant death syndrome	5.1	6	4.3
High-income North America	Female	Sudden infant death syndrome	6.9	8.1	5.8
High-income North America	Male	Meningitis	4.8	5.4	4.3
High-income North America	Female	Meningitis	4.9	5.4	4.4
High-income North America	Male	Encephalitis	2.6	3	2.2
High-income North America	Female	Encephalitis	2.6	3	2.1
High-income North America	Male	Upper respiratory infections	0	0	0
High-income North America	Female	Upper respiratory infections	0	0	0
High-income North America	Male	Otitis media	0	0.1	0
High-income North America	Female	Otitis media	0	0	0
High-income North America	Male	Neonatal disorders	85.7	87.1	84.3
High-income North America	Female	Neonatal disorders	85.7	87.1	84.2
High-income North America	Male	Diarrheal diseases	0.4	0.5	0.3
High-income North America	Female	Diarrheal diseases	0.3	0.4	0.2

High-income North America	Male	Lower respiratory infections	1	1.1	0.9
High-income North America	Female	Lower respiratory infections	1.2	1.3	1
North Africa and Middle East	Male	Upper respiratory infections	0	0	0
North Africa and Middle East	Female	Upper respiratory infections	0	0	0
North Africa and Middle East	Male	Otitis media	0.1	0.5	0
North Africa and Middle East	Female	Otitis media	0	0.1	0
North Africa and Middle East	Male	Neonatal disorders	83.4	86.4	80.4
North Africa and Middle East	Female	Neonatal disorders	79.8	83.2	76.2
North Africa and Middle East	Male	Lower respiratory infections	10.3	12.7	8.2
North Africa and Middle East	Female	Lower respiratory infections	9.7	12.1	7.4
North Africa and Middle East	Male	Meningitis	7.7	10.9	5.3
North Africa and Middle East	Female	Meningitis	5.8	8	4.2
North Africa and Middle East	Male	Encephalitis	4.1	7.3	2
North Africa and Middle East	Female	Encephalitis	3.3	5.5	1.9
North Africa and Middle East	Male	Diarrheal diseases	8	12.1	4.7
North Africa and Middle East	Female	Diarrheal diseases	7	10.9	4.2
North Africa and Middle East	Male	Sudden infant death syndrome	8.8	11.7	6.1
North Africa and Middle East	Female	Sudden infant death syndrome	6	8.1	4.1
Oceania	Male	Otitis media	0	0.1	0
Oceania	Female	Otitis media	0	0	0
Oceania	Male	Meningitis	4.2	7.1	2.2
Oceania	Female	Meningitis	4.6	7.9	2.3
Oceania	Male	Neonatal disorders	75.8	80.1	71.2
Oceania	Female	Neonatal disorders	73.5	79.1	67.2
Oceania	Male	Upper respiratory infections	0	0	0
Oceania	Female	Upper respiratory infections	0.1	0.2	0

Oceania	Male	Sudden infant death syndrome	5.1	9.7	2.1
Oceania	Female	Sudden infant death syndrome	6.6	11.4	3.3
Oceania	Male	Diarrheal diseases	2.2	3.9	1.1
Oceania	Female	Diarrheal diseases	2.4	4.9	0.9
Oceania	Male	Encephalitis	3	5.4	1.6
Oceania	Female	Encephalitis	0.7	1.3	0.3
Oceania	Male	Lower respiratory infections	12.1	16.8	8.3
Oceania	Female	Lower respiratory infections	10	13.6	6.7
South Asia	Male	Lower respiratory infections	23	26.7	19.8
South Asia	Female	Lower respiratory infections	21.6	24.6	18.6
South Asia	Male	Diarrheal diseases	3.5	5.3	2
South Asia	Female	Diarrheal diseases	3.4	5.7	1.8
South Asia	Male	Encephalitis	1.8	2.4	1.3
South Asia	Female	Encephalitis	2.1	2.9	1.5
South Asia	Male	Sudden infant death syndrome	10.6	13.2	8.4
South Asia	Female	Sudden infant death syndrome	11.1	13.6	8.8
South Asia	Male	Upper respiratory infections	0.1	0.2	0
South Asia	Female	Upper respiratory infections	0.1	0.2	0
South Asia	Male	Otitis media	0	0	0
South Asia	Female	Otitis media	0	0.1	0
South Asia	Male	Meningitis	8	10.5	6
South Asia	Female	Meningitis	5	6.6	3.8
South Asia	Male	Neonatal disorders	86.5	88.1	84.6
South Asia	Female	Neonatal disorders	85.4	87.1	83.6
Southeast Asia	Male	Upper respiratory infections	0	0	0
Southeast Asia	Female	Upper respiratory infections	0	0	0

Southeast Asia	Male	Otitis media	0.1	0.1	0
Southeast Asia	Female	Otitis media	0	0	0
Southeast Asia	Male	Neonatal disorders	77.9	80.7	74.8
Southeast Asia	Female	Neonatal disorders	76.3	79	73.3
Southeast Asia	Male	Sudden infant death syndrome	11.9	22.3	6.3
Southeast Asia	Female	Sudden infant death syndrome	12.1	21	7.3
Southeast Asia	Male	Diarrheal diseases	2.5	3.4	1.7
Southeast Asia	Female	Diarrheal diseases	2.3	3.5	1.4
Southeast Asia	Male	Meningitis	3.9	5	3.1
Southeast Asia	Female	Meningitis	4.2	5.4	3.2
Southeast Asia	Male	Encephalitis	1.7	2.5	0.5
Southeast Asia	Female	Encephalitis	0.7	0.9	0.6
Southeast Asia	Male	Lower respiratory infections	7.3	9	5.9
Southeast Asia	Female	Lower respiratory infections	7.2	8.5	6
Southern Latin America	Male	Diarrheal diseases	1.7	2.6	1
Southern Latin America	Female	Diarrheal diseases	1.9	2.9	1.2
Southern Latin America	Male	Meningitis	2.8	3.7	2.1
Southern Latin America	Female	Meningitis	3.1	4	2.3
Southern Latin America	Male	Encephalitis	0.9	1.4	0.6
Southern Latin America	Female	Encephalitis	1	1.4	0.7
Southern Latin America	Male	Sudden infant death syndrome	6.6	9.4	4.4
Southern Latin America	Female	Sudden infant death syndrome	8.1	11.6	5.2
Southern Latin America	Male	Lower respiratory infections	1	1.3	0.8
Southern Latin America	Female	Lower respiratory infections	1.2	1.5	0.9
Southern Latin America	Male	Upper respiratory infections	0	0	0
Southern Latin America	Female	Upper respiratory infections	0	0	0

Southern Latin America	Male	Otitis media	0	0.1	0
Southern Latin America	Female	Otitis media	0	0	0
Southern Latin America	Male	Neonatal disorders	79.3	81.6	76.5
Southern Latin America	Female	Neonatal disorders	79.5	81.7	77.2
Southern Sub-Saharan Africa	Male	Lower respiratory infections	6.8	8.6	5.3
Southern Sub-Saharan Africa	Female	Lower respiratory infections	8.1	10.2	6.4
Southern Sub-Saharan Africa	Male	Sudden infant death syndrome	7.7	11	5.1
Southern Sub-Saharan Africa	Female	Sudden infant death syndrome	6.9	10.3	4.8
Southern Sub-Saharan Africa	Male	Diarrheal diseases	1.7	2.5	1.1
Southern Sub-Saharan Africa	Female	Diarrheal diseases	1.7	2.7	1
Southern Sub-Saharan Africa	Male	Encephalitis	1.6	2.6	1
Southern Sub-Saharan Africa	Female	Encephalitis	1.9	3.5	1.1
Southern Sub-Saharan Africa	Male	Upper respiratory infections	0.3	0.7	0.2
Southern Sub-Saharan Africa	Female	Upper respiratory infections	0.3	0.6	0.1
Southern Sub-Saharan Africa	Male	Otitis media	0	0.1	0
Southern Sub-Saharan Africa	Female	Otitis media	0	0	0
Southern Sub-Saharan Africa	Male	Meningitis	3.5	5.1	2.6
Southern Sub-Saharan Africa	Female	Meningitis	3.8	5.6	2.7
Southern Sub-Saharan Africa	Male	Neonatal disorders	88.6	90	87.1
Southern Sub-Saharan Africa	Female	Neonatal disorders	87.1	88.8	85.1
Tropical Latin America	Male	Otitis media	0.2	0.4	0.1
Tropical Latin America	Female	Otitis media	0.1	0.2	0
Tropical Latin America	Male	Meningitis	1.7	2	1.4
Tropical Latin America	Female	Meningitis	1.2	1.4	1.1
Tropical Latin America	Male	Neonatal disorders	73.1	74.9	71.1
Tropical Latin America	Female	Neonatal disorders	72.7	74.8	70.2

Tropical Latin America	Male	Upper respiratory infections	0.1	0.1	0
Tropical Latin America	Female	Upper respiratory infections	0.1	0.1	0
Tropical Latin America	Male	Diarrheal diseases	0.8	1.1	0.6
Tropical Latin America	Female	Diarrheal diseases	0.5	0.7	0.4
Tropical Latin America	Male	Encephalitis	2.2	3.4	1.7
Tropical Latin America	Female	Encephalitis	2.3	3.7	1.7
Tropical Latin America	Male	Lower respiratory infections	2.1	2.6	1.8
Tropical Latin America	Female	Lower respiratory infections	1.9	2.3	1.6
Tropical Latin America	Male	Sudden infant death syndrome	6.5	7.8	5.4
Tropical Latin America	Female	Sudden infant death syndrome	5	6	4.2
Western Europe	Male	Upper respiratory infections	0	0	0
Western Europe	Female	Upper respiratory infections	0	0	0
Western Europe	Male	Otitis media	0	0.1	0
Western Europe	Female	Otitis media	0	0.1	0
Western Europe	Male	Neonatal disorders	78.6	80.4	76.8
Western Europe	Female	Neonatal disorders	78.1	79.9	76.2
Western Europe	Male	Lower respiratory infections	0.8	1	0.6
Western Europe	Female	Lower respiratory infections	1.1	1.3	0.9
Western Europe	Male	Diarrheal diseases	0.6	0.8	0.4
Western Europe	Female	Diarrheal diseases	0.6	0.8	0.4
Western Europe	Male	Meningitis	4.3	5.2	3.5
Western Europe	Female	Meningitis	4.8	5.8	4
Western Europe	Male	Encephalitis	1.4	1.8	1.1
Western Europe	Female	Encephalitis	1.4	1.7	0.9
Western Europe	Male	Sudden infant death syndrome	6	7.3	4.9
Western Europe	Female	Sudden infant death syndrome	7.1	8.7	5.8

Western Sub-Saharan Africa	Male	Sudden infant death syndrome	5.2	8.5	3.5
Western Sub-Saharan Africa	Female	Sudden infant death syndrome	5.1	8.7	3.4
Western Sub-Saharan Africa	Male	Meningitis	4.5	5.5	3.7
Western Sub-Saharan Africa	Female	Meningitis	4.6	5.6	3.7
Western Sub-Saharan Africa	Male	Encephalitis	2.5	3.5	1.1
Western Sub-Saharan Africa	Female	Encephalitis	3.9	5.4	1.9
Western Sub-Saharan Africa	Male	Upper respiratory infections	0.2	0.4	0
Western Sub-Saharan Africa	Female	Upper respiratory infections	0	0	0
Western Sub-Saharan Africa	Male	Otitis media	0.1	0.8	0
Western Sub-Saharan Africa	Female	Otitis media	0.1	0.3	0
Western Sub-Saharan Africa	Male	Neonatal disorders	85.1	87	82.9
Western Sub-Saharan Africa	Female	Neonatal disorders	82.2	84.4	79.7
Western Sub-Saharan Africa	Male	Diarrheal diseases	2.8	3.5	2
Western Sub-Saharan Africa	Female	Diarrheal diseases	2.6	3.3	1.8
Western Sub-Saharan Africa	Male	Lower respiratory infections	12.4	14.4	10.8
Western Sub-Saharan Africa	Female	Lower respiratory infections	11.6	13.5	10

PBLBW= preterm birth and low birth weight; DALY= disability-adjusted life year; GBD= Global Burden of Disease.
